# The module triad: a novel network biology approach to utilize patients’ multi-omics data for target discovery in ulcerative colitis

**DOI:** 10.1038/s41598-022-26276-x

**Published:** 2022-12-15

**Authors:** Ivan Voitalov, Lixia Zhang, Casey Kilpatrick, Johanna B. Withers, Alif Saleh, Viatcheslav R. Akmaev, Susan Dina Ghiassian

**Affiliations:** Scipher Medicine Corporation, 221 Crescent St Suite 103A, Waltham, MA 02453 USA

**Keywords:** Network topology, Complex networks, Target identification, Ulcerative colitis

## Abstract

Tumor necrosis factor-$\alpha$ inhibitors (TNFi) have been a standard treatment in ulcerative colitis (UC) for nearly 20 years. However, insufficient response rate to TNFi therapies along with concerns around their immunogenicity and inconvenience of drug delivery through injections calls for development of UC drugs targeting alternative proteins. Here, we propose a multi-omic network biology method for prioritization of protein targets for UC treatment. Our method identifies network modules on the Human Interactome—a network of protein-protein interactions in human cells—consisting of genes contributing to the predisposition to UC (Genotype module), genes whose expression needs to be modulated to achieve low disease activity (Response module), and proteins whose perturbation alters expression of the Response module genes to a healthy state (Treatment module). Targets are prioritized based on their topological relevance to the Genotype module and functional similarity to the Treatment module. We demonstrate utility of our method in UC and other complex diseases by efficiently recovering the protein targets associated with compounds in clinical trials and on the market . The proposed method may help to reduce cost and time of drug development by offering a computational screening tool for identification of novel and repurposing therapeutic opportunities in UC and other complex diseases.

## Introduction

Ulcerative colitis (UC) is a complex disease characterized by chronic intestinal inflammation and is thought to be caused by an abnormal immune response to intestinal microbiota in genetically predisposed patients^1^. Standard treatment of UC includes aminosalicylates and steroids^2^ and, if low disease activity is not achieved, biologics such as tumor necrosis factor-$\alpha$ inhibitors (TNFi) are recommended^3^. Nonetheless, about 40% of patients are unresponsive to TNFi treatment^4^, and up to 10% of initial responders will lose their response to TNFi therapy each year^3^. These difficulties with TNFi therapies along with financial incentives led to research and development of alternative therapeutic approaches: JAK inhibitors^5^, IL-12/IL-23 inhibitors^6^, S1P-receptor modulators^7^, anti-integrin agents^8^, and novel TNFi compounds^3^. These approaches target biological mechanisms contributing to aberrant immune response, requiring detailed knowledge about UC pathogenesis. However, due to concerns around immunogenicity and inconvenience of drug delivery through injections, there is an increasing interest in development of additional orally administered small molecule drugs.

Development of novel drugs requires identification of molecular targets whose modulation leads to low disease activity or remission. With the surge in multi-omic data, machine learning (ML) and artificial intelligence (AI) became widely used for many tasks in therapeutics such as target prioritization, drug design, drug target interaction prediction and small molecule optimization^9^. Current ML/AI approaches for target prioritization primarily focus on search for genes that are involved in a given disease. These genes may be inferred by training classifiers using features constructed from the disease-specific gene expression and mutation data, along with information about relevant protein-protein, metabolic, and transcriptional interactions^10–13^, or by analyzing existing textual databases and research literature for disease-genes associations using natural language processing (NLP) methods^14,15^.

Yet, many ML/AI approaches suffer from exploration biases and data incompleteness^16,17^. Moreover, systematic analyses demonstrated that drugs approved by the U.S. Food and Drug Administration (FDA) do not directly target protein products of the disease-associated genes^18,19^. Network-based target prioritization methods address these issues by aggregating proteomic, metabolomic, and transcriptomic interactions as well as associations between drugs, diseases, and genes in the form of networks, and deriving the network-based features distinguishing feasible targets in an unbiased and unsupervised manner^19–22^. Nonetheless, there is not yet a network-based framework that simultaneously captures the relation between disease formation and successful treatment as a means to identify novel potential targets.

To address this, here we propose a network-based approach for target prioritization for UC that utilizes three network regions (modules) of the Human Interactome (HI)—a network of protein-protein interactions in human cells—that we refer to as the *module triad*:

1. *Genotype module*—a set of genes associated to the genetic predisposition of UC;
2. *Response module*—a set of genes whose expression needs to be altered in order to achieve low disease activity;
3. *Treatment module*—a set of proteins that need to be targeted to alter expression of Response module genes in a direction to achieve low disease activity.

We hypothesize that feasible targets should simultaneously (a) be *topologically relevant* to the Genotype module, i.e., be in the network vicinity of the genes associated with a particular disease, following the findings in^19^, and (b) be *functionally similar* to the Treatment module, i.e., have a similar transcriptomic downstream effects to that of the Treatment module proteins upon their perturbation. We demonstrate the utility of the proposed framework using UC as a showcase by efficiently recovering known targets approved for UC, and distinguishing targets being at different stages of development for UC based on the network-derived rankings. To the best of our knowledge, the module triad framework is the first attempt to connect biological mechanisms underlying complex disease development and its treatment dynamics from the network perspective. Additionally, we validate the module triad approach on 3 other diseases (psoriasis, Pakinson’s disease and Alzheimer’s disease) to demonstrate that the proposed framework is directly extendable to other complex diseases with known gene-disease associations, available gene expression data of patients before and after treatment, and perturbation experiments in appropriate cell lines.

## Results

### Overview of the module triad target prioritization framework

The module triad framework consists of the two main parts: (1) discovery of the module triad for a given disease, and (2) novel target discovery based on the identified module triad (Fig. 1).

For discovery of the module triad, each module is mapped to the HI using auxiliary disease-specific information. The Genotype module is constructed by analyzing gene-disease associations databases to locate genes whose mutations predetermine the formation of the disease phenotype. The Response module consists of the genes that are significantly down-/up-regulated after treatment in patients that achieved low disease activity. Treatment module construction is a two-step procedure. First, we use the LINCS L1000 perturbations database^23^ to identify small molecule compounds that result in gene expression profiles similar to that observed for Response module genes after treatment. Second, we use DrugBank^24^ and Repurposing Hub^25^ databases to extract the set of proteins targeted by these compounds; these proteins are mapped to the HI resulting in the Treatment module.

All proteins (nodes) of the HI are then ranked based on the constructed Genotype and Treatment modules. For each node, its topological relevance to the Genotype module is assessed based on its *proximity*^19^ which is computed based on the average shortest distance from the node to the Genotype module nodes. Functional similarity of the node to the Treatment module is assessed using *selectivity* which is computed based on the average diffusion state distance (DSD)^26^ of the node to the Treatment module nodes. For details on computing proximity and selectivity, see Fig. 1 and "Methods". Finally, all HI nodes are ranked based on their proximity and selectivity scores, and these two rankings are merged into a single combined rank using the rank product^27^.

**Figure 2:**
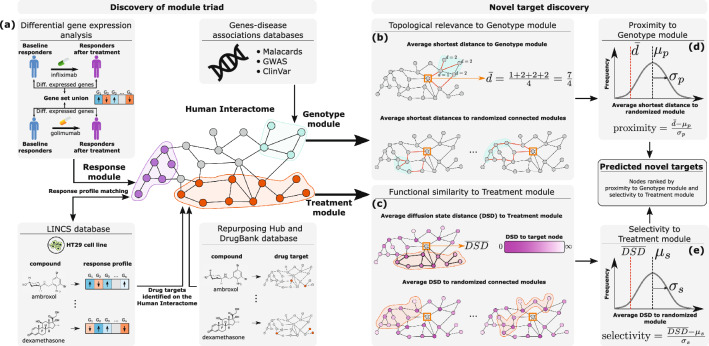
Overview of the module triad framework. **(a)** The pipeline for discovery of the UC module triad on the Human Interactome: the Response module is derived from differentially expressed genes before and after treatment in the patients with active UC who responded to TNFi therapies (infliximab and golimumab); the Genotype module is derived by mapping the genes associated with UC on the Human Interactome; the Treatment module is derived by selecting the small molecule compounds resulting in the alteration of gene expression of the Response module genes using experimental data in the HT29 cell line and mapping the compounds to their protein targets. Target prioritization based on the discovered module triad: **(b),(d)** topological relevance of a node to the Genotype module is measured by computing the average shortest path length of the node to all Genotype module nodes, and comparing it to the empirical distribution of average shortest path lengths to the randomized connected subnetworks of the same size as the Genotype module using *Z*-score (proximity); **(c),(e)** functional similarity of a node to the Treatment module is measured by computing the average diffusion state distance (DSD) of the node to all Treatment module nodes, and comparing it to the empirical distribution of average DSDs to the randomized connected subnetworks of the same size as the Treatment module using *Z*-score (selectivity). All nodes are ranked based on proximity and selectivity, and their ranks are combined using rank product to obtain the final target ranking.

### UC genotype module

The protein products of genes associated with a disease usually are not randomly scattered on the HI, but rather form clusters of interconnected nodes^28–31^ reflecting the existence of an underlying biological mechanism behind disease formation. Studying network properties of these interconnected clusters has advanced understanding of disease molecular mechanisms, target discovery and drug repurposing^17,19,32,33^.

To include the notion of UC genetic associations in the module triad framework, we use the GWAS Catalog^34^, ClinVar^35^, and MalaCards^36^ databases to extract genes reported to have associations with UC (see "Methods" for details). We find a total of 194 genes reported in at least one of the three databases as being associated with UC, and 174 of them ($89.7 \%$) are mapped to their corresponding protein products in the HI. As expected, the protein products are not randomly scattered on the network; $64.9 \%$ (113/174) of proteins are interconnected, forming a largest connected component (LCC) that is significantly larger than expected at random ($Z\text {-score} = 4.82$, $p < 10^{-4}$). We define this LCC as the Genotype module representing the genetic predispositions to UC. Following the work in^19^, we assume that a feasible target should be located in the topological vicinity of the Genotype module.

### Successful UC treatment is reflected at the transcriptomic level

Besides being topologically close to the genes leading to predisposition to UC, a feasible target should also be functionally relevant to the treatment of UC. Specifically, we assume that UC treatment dynamics is reflected at the transcriptomic level, and perturbing a feasible target should result in transcriptional changes similar to that observed upon successful UC treatment.

To test if UC treatment is indeed reflected at the transcriptomic level, we collect the gene expression data of normal tissue controls and patients with active UC undergoing treatment with TNFi drugs—either infliximab or golimumab—from several studies^37–43^ (see Supplementary Table S1 for details). Next, we identify a set of 545 genes that are differentially expressed between patients with active UC and normal controls. We use these genes as features for Uniform Manifold Approximation and Projection (UMAP) embedding^44^ of the gene expression profiles of normal controls and UC patients before and after treatment, split into two groups: patients who achieved low disease activity after treatment (*responders*) and those who did not (*non-responders*), Fig. 2.

From UMAP embedding, we do not observe any apparent distinction between the pre-treatment gene expression profiles of responders and non-responders to infliximab or golimumab. Additionally, we do not find any differentially expressed genes between the pre-treatment gene expression profiles of responders and non-responders (see Supplementary Note S1.1). Conversely, the post-treatment gene expression profiles of responders are clustered closely with those of normal controls, whereas post-treatment profiles of non-responders to infliximab or golimumab are clustered separately from those of normal controls, indicating that gene expression profiles with high similarity to those of normal controls may be reflective of successful UC treatment. Motivated by these observations, we define *“molecular response”* to UC treatment as reversal of the gene expression profile of UC patients upon treatment to resemble the gene expression profiles of normal controls.

**Figure 3:**
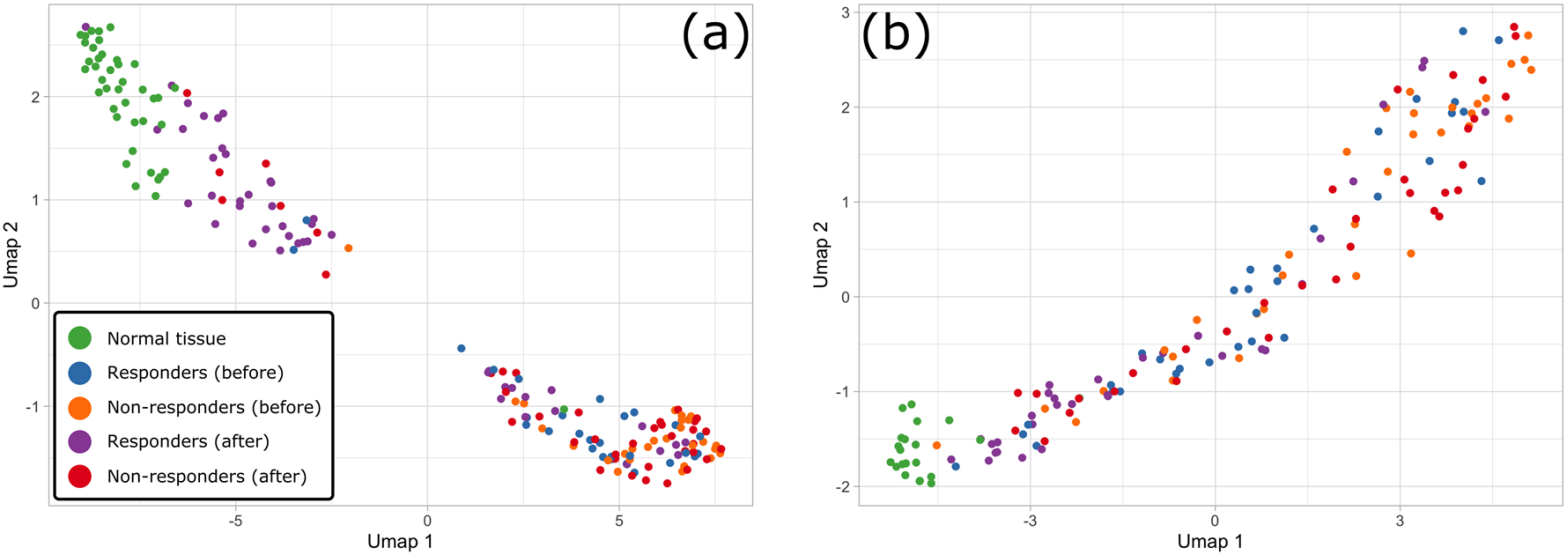
Gene expression profiles of normal tissue controls and UC active patients before and after TNFi therapy. The first two coordinates of the UMAP embedding of gene expression profiles are based on the set of 545 differentially expressed genes between patients with active UC and normal controls for **(a)** infliximab TNFi treatment; **(b)** golimumab TNFi treatment.

### UC Response module

To further understand what transcriptional changes cause responders’ gene expression profiles to become more similar to those of normal controls, we perform differential expression analysis of pre- and post-treatment gene expression profiles of responders. We find that only a small fraction of genes dysregulated in responders before treatment with respect to normal controls exhibits significant changes in expression after treatment (see Supplementary Note S1.1). The expression of these genes is reverted in responders upon treatment, i.e., genes down-regulated in responders before treatment with respect to normal controls become up-regulated after treatment, and vice versa. Yet, these transcriptional changes are sufficient to make the gene expression profiles of responders and normal controls similar based on the profile embeddings shown in Fig. 2, and are indicative of patients who achieved low disease activity following treatment. For brevity, we call this set of genes indicative of molecular response to UC treatment the *RBA (responders before-after)* set. We construct the RBA set specific to TNFi treatment of UC by taking the union of RBA genes determined from the infliximab- and golimumab-based studies (see "Methods").

We hypothesize that genes belonging to the RBA set are related to each other via one or multiple biological pathways, proper functioning of which is restored by inhibition of TNF-$\alpha$, and therefore we expect them to be located close to each other on the HI. To test this, we map TNFi RBA genes on the HI to construct a subnetwork comprised of the nodes corresponding to the RBA genes. The RBA set forms a significant LCC on the HI (91 out of 271 nodes, $34 \%$) as compared to a randomly selected set of nodes with preserved degree sequence ($Z\text {-score} = 9.24$, $p < 10^{-4}$). This refined set of genes in the RBA LCC is defined as the Response module, i.e., the region of the HI transcriptionally altered when a UC patient achieves low disease activity in response to therapeutic intervention.

### UC Treatment module

We find that successful treatment of UC requires reverting the expression profile of the Response module nodes by studying the gene expression profiles of UC patients undergoing TNFi therapies. We further hypothesize that inhibition of TNF-$\alpha$ is not the only way to achieve desirable transcriptomic effects in the Response module genes, and perturbation of other proteins may achieve similar downstream effects.

We test this hypothesis by analyzing alternative perturbations that are experimentally validated to result in a molecular response similar to the one observed upon successful TNFi therapy. We consider differential gene expression effects (signatures) resulting from perturbation of human cell lines with small molecule compounds obtained from the LINCS L1000 project^23^. Perturbation signatures are derived from LINCS L1000 Level 5, Phase I data containing gene-wise *Z*-scores that indicate the magnitude and direction of change in gene expression for 14,513 compound experiments in the HT29 cell line (human colorectal adenocarcinoma cell line). We consider perturbation experiments in the HT29 cell line because of its relevance to UC-affected tissue (colon) and relatively wide coverage of small molecule compounds.

To find the compounds and corresponding target proteins that revert expression of the Response module genes, we assess the LINCS L1000 experiments by computing the Weighted Connectivity Score (WTCS)^23^ with respect to the up- and down-regulated genes in the Response module using gene-wise perturbation *Z*-scores for each HT29 cell line experiment. To assess statistical significance of the WTCS for a given experiment, we employ a randomization procedure assigning a pair of *p*-values, $p_{up}$ and $p_{down}$, associated with the enrichment scores of the up- and down-regulated genes (see "Methods"). Compound experiments that have $p_{up} \ge 0.05$ and $p_{down} \ge 0.05$, and $WTCS \ge 0$ are excluded. This filtering ensures consideration of compounds that have a positive and significant therapeutic effect in terms of reverting the expression of Response module genes.

Of 14,513 compound experiments conducted in the HT29 cell line, 68 experiments have a statistically significant WTCS, ranging from $-0.642$ to $-0.480$. 69 proteins appear as a target for at least one of the 25 unique compounds evaluated in these 68 experiments, according to DrugBank and Repurposing Hub databases. We find that two proteins could not be mapped to the HI (i.e., they have no known protein interaction partners), and 43 out of 67 remaining proteins ($64\%$) form an LCC of significant size ($Z-\text {score} = 3.39$, $p < 10^{-4}$). We call this LCC the Treatment module.

Given the derived module triad for UC, we observe that 2 genes belonging to the Treatment module also belong to the Genotype module (TNF-$\alpha$, AR), and none of the Treatment module genes belong to the Response module, while the Genotype and Response modules share 7 common genes (IL1RN, CXCL8, HLA-DRA, IL1B, FCGR2A, IL7R, ICAM1), Supplementary Fig. S4. As expected, one of the targets belonging to the Treatment module is TNF-$\alpha$. Moreover, by construction, targeting proteins belonging to the Treatment module results in transcriptional changes within the Response module similar to those observed upon successful TNFi therapy. Hence, proteins belonging to the Treatment module offer intervention opportunities for treating UC patients.

### Target ranking

Besides potential intervention opportunities suggested directly from the Treatment module nodes, the Genotype and Treatment modules can be used to prioritize, in an unsupervised fashion, all nodes in the HI for their potential as a UC treatment target. We hypothesize that a feasible target should simultaneously satisfy the following two network properties. First, it must be topologically close to HI nodes associated with genetic predisposition to UC (Genotype module). Target prioritization based on the network proximity of nodes to disease modules is predictive of therapeutic effects of drugs with known targets across multiple diseases^19^. Therefore, to quantify topological relevance of a given HI node to the UC Genotype module, we calculate its proximity to the Genotype module based on the average network shortest path of the node to the Genotype module (see "Methods").

Second, targeting a feasible target should cause transcriptional changes similar to those observed upon successful UC treatment. The Treatment module defines a network region consisting of nodes that, upon perturbation, result in a desirable transcriptional changes in Response module genes. Therefore, proteins that are functionally similar to Treatment module proteins are also promising targets. Yet, to find such targets, we need a methodology to quantify downstream transcriptional effect similarities of HI nodes based on network structure. For this task, we use diffusion state distance (DSD)^26^—a metric based on network random walks designed to capture propagation-based topological similarities between each pair of nodes in the network—because of its superior performance in predicting protein functional annotations^26^.

To evaluate whether DSD reflects similarities in downstream transcriptional effects between different proteins, we analyze the recovery of approved drugs for six complex diseases (Alzheimer’s disease, ulcerative colitis, rheumatoid arthritis, multiple sclerosis, psoriasis and Parkinson’s disease) based only on DSD between the HI nodes (see "Methods"). The targets of each approved drug should result in similar therapeutic effects of treating a given disease. Thus, one should be able to efficiently recover approved targets by knowing only one drug target and its DSD to other HI nodes. We perform such target recovery separately for each approved target and complex disease to derive receiver operator characteristic (ROC) and Precision-Recall (PR) curves (Supplementary Fig. S5). We observe that knowing DSD from an approved drug target to the rest of the nodes in the HI is sufficient to recover the rest of the known approved targets in each complex disease.

Yet, a node that has low DSD to the Treatment module may be equally close to other randomly chosen modules of equal size in the HI. To account for this, we quantify functional similarity between HI nodes and the Treatment module using *selectivity*—a network-based measure based on the DSD that considers statistical significance of the DSD between a node and a given network module (see "Methods").

Finally, we rank all HI nodes based on their proximity to the Genotype module and selectivity to the Treatment module, and use the rank product^27^ to determine the final combined ranking of the nodes (see "Methods").

### *In silico* validation of the UC module triad target prioritization

To test if the proposed target ranking yields meaningful results, we obtain drug targets approved for UC treatment from the Citeline database^45^ (see "Methods"). The resulting list consists of 23 targets mapped on the HI. We observe that the approved targets are simultaneously highly proximal to the Genotype module and selective to the Treatment module compared to the rest of HI nodes (Fig. 3, panel (a)).

We validate the proposed target prioritization method by ranking all nodes in the HI according to their proximity to the Genotype module, selectivity to the Treatment module, and combination of both ranks. We expect that a well-performing target prioritization method would assign high ranks to the protein targets approved for UC treatment. We assess the rankings by constructing ROC curves for each ranking and measuring the area under them (AUC). In our validation, we treat the approved targets as a positive class, while the rest of nodes as a negative class, which leads to highly imbalanced set. To address this issue, we also employ a precision-recall (PR) curve and area under it (AUPR) which is known to be a more informative measure in the case of highly imbalanced classes^46^, as well as measure precision and recall separately at top-500 (approximately top-$3\%$) ranked nodes.

To compare performance of the proposed network measures for the task of target prioritization, we check performance of additional three methods widely employed for prioritizing proteins based on network information: *Local radiality (LR)*^21^, *random walk with restart (RWR)*^47,48^, and *node2vec* embedding^48,49^. All three methods are unsupervised and are based on the network information. At a high level, LR additionally employs information about transcriptomic changes observed in a disease (represented in our framework by the Response module), while both RWR and node2vec may rank network nodes with respect to a set of predefined “seed” nodes. As genes associated to disease are often used as candidate targets for therapeutic intervention^50,51^, we run both RWR and node2vec algorithms using all genetic associations for UC as a set of seed nodes. We provide more details on each of the reference prioritization algorithms in "Methods".

We report all performance metrics for UC target recovery in Table 1. We observe that while both proximity and selectivity efficiently recover known approved targets on their own, a combination of both performs better in terms of AUC suggesting a synergistic effect of these network measures for target prioritization (Fig. 3, panel (b)). Other target prioritization methods (LR, RWR, node2vec) also efficiently recover the approved UC targets, albeit less efficiently than the module triad prioritization. We observe that in terms of AUPR, Precision@500 and Recall@500 the best performing method is selectivity to the Treatment module, suggesting that adding phenotype information to the network view of the UC formation and treatment is beneficial for more efficient recovery of the approved targets. Recall values corresponding to approved UC target recovery at different top-*K* levels for all tested methods are reported in Supplementary Table S3.

In addition to using Citeline-reported approved targets for UC as a positive set, we also report performance of our method and other network-based target prioritization methods using the approved targets derived from the three open access databases: Repurposing Hub^25^, DrugBank^24^, and Therapeutic Target Database^52^. For all approved drugs indicated for UC in these databases, we extract their known targets and assume them to be positive set for UC. We note that the target sets derived from the three open access databases differ (see Supplementary Fig. S6), therefore, we consider two approaches of combining the targets from the three target sets: (1) union of all three sets, i.e., all targets that are listed in at least one database as approved for UC; (2) targets that are listed in at least two out of three databases. Performance metrics are reported in Table 1, and ROC/PR curves for all considered positive sets are shown in Supplementary Fig. S6. We observe that in this setting the combined ranking and selectivity-based rankings are most efficient in recovering the approved UC targets, followed by RWR in the case of Precision@500 and Recall@500 metrics.

Finally, we hypothesize that drugs that are under consideration as a UC treatment (i.e., being tested in clinical and preclinical trials) should target nodes that have a lower combined ranking based on the proximity and selectivity when compared to the targets that are already launched for UC. This is because launched targets have already been assessed through clinical stages for their ability to ameliorate disease activity in UC patients, while targets that are not yet launched may not necessarily be efficacious for treatment of UC. To test this, we compare the distribution of the combined ranks for the targets of drugs that are launched, in clinical trials (Phase I, II, III), or preclinical studies, as reported by Citeline (Fig. 3, panel (c)). As expected, the median combined ranking of the targets corresponding to the launched drugs is higher, followed by those in clinical trials, then in preclinical studies.

**Figure 4:**
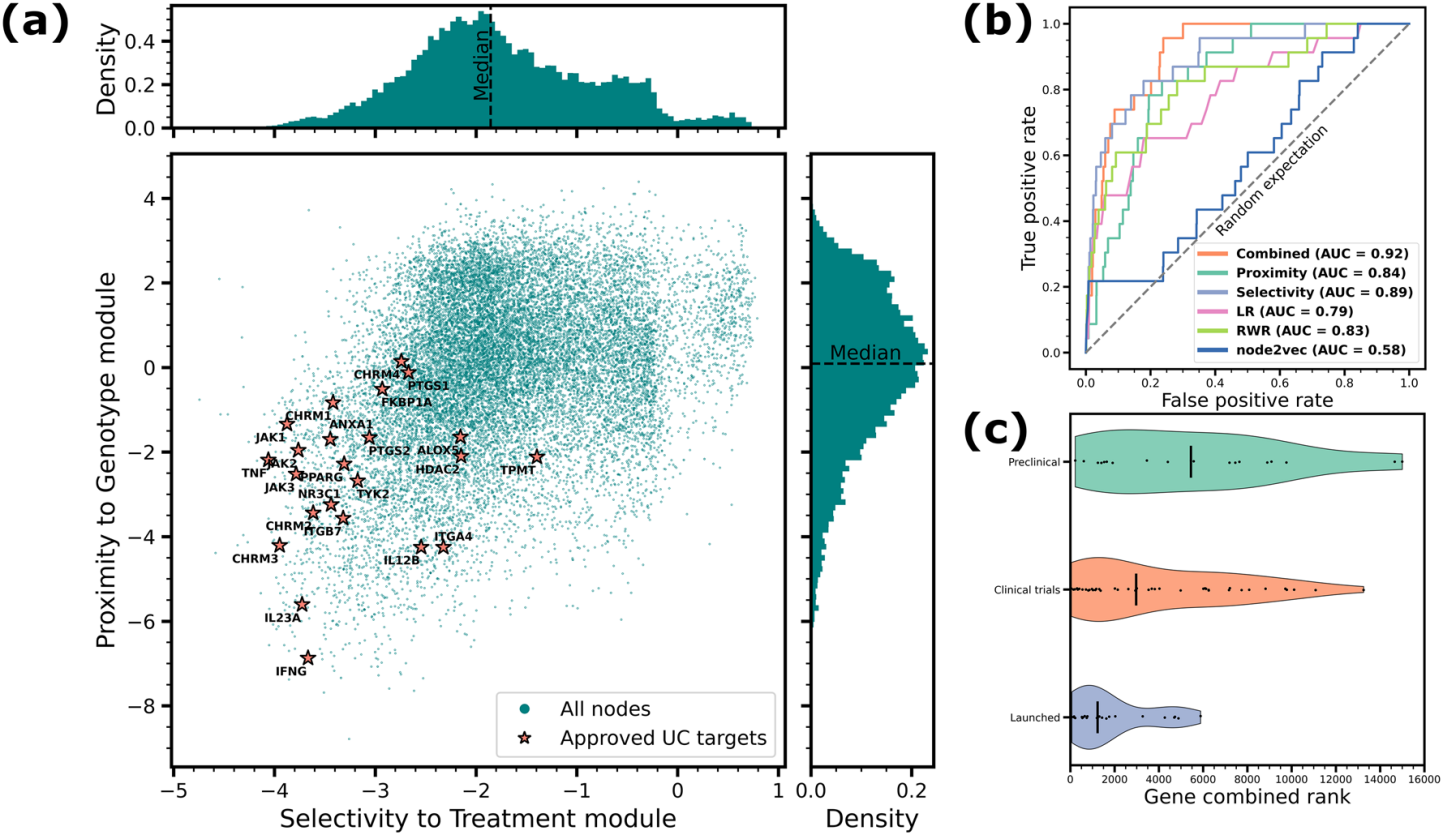
*In silico* validation of the module triad target prioritization. **(a)** Selectivity-proximity scatter plot of the HI nodes with 23 targets approved for UC treatment highlighted. More selective and proximal targets are located towards the lower left of the scatter plot. **(b)** Receiver operator characteristic (ROC) curves for recovery of the approved UC targets using proximity to the Genotype module, selectivity to the Treatment module, a combination of both, the Local radiality (LR) with respect to the Response module, random walk with restart (RWR) and node2vec embedding with respect to all UC disease associations, with corresponding areas under the curve (AUC). **(c)** Violin plots of the combined selectivity-proximity ranks of the targets launched for UC, and targets being at preclinical and clinical trials development stage for UC.

**Table 1:** Performance of the proposed module triad method and other network-based target prioritization methods on recovery of known approved targets in ulcerative colitis.

	Module triad prioritization			Reference prioritization methods		
Performance metric	Combined (sel. & prox.)	Selectivity to TM	Proximity to GM	Local radiality	Random walk	node2vec
**Citeline,** ${\textbf{23}}$** approved targets**						
AUC	$\mathbf {0.92}$	0.89	0.84	0.79	0.83	0.58
AUPR	0.0159	$\mathbf {0.0211}$	0.0068	0.0108	0.0184	0.0084
Precision@500	0.016	$\mathbf {0.022}$	0.004	0.016	0.016	0.010
Recall@500	0.3478	$\mathbf {0.4783}$	0.0870	0.3478	0.3478	0.2174
**Open access databases (union),** ${\textbf{63}}$ **approved targets**						
AUC	$\mathbf {0.72}$	0.69	0.68	0.61	0.61	0.56
AUPR	0.0120	$\mathbf {0.0156}$	0.0071	0.0075	0.0095	0.0063
Precision@500	0.020	$\mathbf {0.028}$	0.006	0.014	0.012	0.010
Recall@500	0.1639	$\mathbf {0.2295}$	0.0492	0.1148	0.0984	0.0820
**Open access databases (at least two)**, $\textbf{7}$ **approved targets**						
AUC	$\mathbf {0.85}$	0.79	0.81	0.80	0.83	0.60
AUPR	$\mathbf {0.0102}$	0.0052	0.0072	0.0084	0.0101	0.0057
Precision@500	0.004	$\mathbf {0.006}$	0.002	0.004	$\mathbf {0.006}$	0.004
Recall@500	0.2857	$\mathbf {0.4286}$	0.1429	0.2857	$\mathbf {0.4286}$	0.2857

Known approved targets are derived from Citeline database, as well as the three open access databases. Best performing methods are highlighted in bold.

### Pathway analysis of the UC prioritized targets

In addition to *in silico* validation of our model using approved drug targets, we investigate the biological function of the top-$1\%$ of predicted targets for their role in UC. We perform an overrepresentation analysis to determine if pathways are represented in the target gene set using Reactome^53^. We identify 70 pathways that are significantly enriched within the gene set (hypergeometric test, $p < 0.05$), and 7 pathways that pass the FDR-corrected significance threshold of 0.05, see Supplementary Table S5 for details. The pathways with the highest number of total entities found are generally related to cytokine signaling and signaling by interleukins^54^.

We identify several specific cytokine pathways that play an important role in UC. We find significant enrichment in pathways related to interleukin-10 (IL-10), IL-4 and IL-13 signaling^55–57^. IL-10 is a prominent anti-inflammatory cytokine with promotor polymorphisms that can alter the serum level of IL-10 in patients with inflammatory bowel disease (IBD)^58,59^. Clinical trials for recombinant human IL-10 (Tenovil) found that patients with Crohn’s Disease (CD) who had high disease burden and low IL-10 levels could benefit from IL-10 supplementation. Overall, the combined IL-10 CD cohorts did not reach statistical significance for improved disease outcome measures, highlighting the individual level disease heterogeneity^60^. Despite promising preclinical results in animal models of colitis, IL-10 remains an unexplored therapeutic target in UC^61–63^. Another cytokine enriched pathway involves IL-4 and IL-13. IL-4 and IL-13 are T helper-(Th) type 2 cytokines that mediate B and T cell inflammation in UC^64^. Both IL-4 and IL-13 expression is higher in patients with UC compared to CD and contributes to impaired epithelial barrier function^65,66^. Interferon-$\beta$-1a administration in patients with UC inhibits IL-13 production from natural killer T-cells and is associated with clinical improvement, highlighting the potential for IL-13 as a target^67^.

The homeostasis between intestinal microflora and the gut immune system have been implicated in the development of IBD. One of the top pathways enriched in the UC targets represent the Dectin-1 (CLEC7A) inflammasome pathway. The Dectin-1 recognizes glucans found on fungi and activates inflammatory cytokine production through T helper 17 (Th17) cells^68^. Polymorphisms in CLEC7A have been associated with medically (treatment) refractory UC populations but not as a susceptibility gene indicating it might be a good target for severe disease populations^69^. Preclinical mouse models with CLEC7A knock-out or treated with Dectin-1 antagonists suppress the development of colitis through modulating Treg differentiation by modifying the microbiota in the gut^70^.

Unsurprisingly, another highly enriched target pathway is the FOXP3 and RUNX1 regulatory T cell (Treg) pathway. Tregs are an important immunomodulatory balance point serving to mitigate the peripheral immune response. Altered balance between Foxp3$^{+}$ CD4$^{+}$ Treg cells and effector T cells in the intestinal mucosa contributes to the development of IBD^71^. Interestingly, CD4$^{+}$CD8$^{+}$ $\alpha \alpha$ colonic Tregs secrete IL-10 in response to intestinal bacteria and are reduced in IBD^72^. Given that our top UC targets are implicated in IL-10 signaling, microbial homeostasis and Treg differentiation it highlights the utility of using protein-protein interaction networks to identify novel insights.

### Module triad prioritization for psoriasis, Parkinson’s disease and Alzheimer’s disease

To demonstrate how the proposed target prioritization pipeline generalizes to other diseases, we repeat the validation using the approved targets in three additional complex diseases—psoriasis, Parkinson’s disease (PD) and Alzheimer’s disease (AD). We select these diseases from the set of complex diseases that we used to illustrate the utility of the DSD metric in recovery of known approved targets above, while omitting rheumatoid arthritis (RA) and multiple sclerosis (MS) as including them would require laborious data collection and normalization. Details on the choice of complex diseases and module triad construction for psoriasis, PD and AD are outlined in Supplementary Note S1.2.

For validation, we consider the three sets of approved targets, similar to the UC case: approved targets according to the Citeline database, union of the targets reported by the three open access databases, and the targets reported in at least two of the public access databases. Performance metrics for psoriasis, PD and AD targets are reported in Table 2, and more detailed performance plots are shown in Supplementary Figure S7, S8, S9.

In case of psoriasis, we observe that RWR prioritization outperforms other algorithms in all metrics except for AUC for the set of Citeline-derived approved targets, our target prioritization method outperforms the rest of algorithms on the union set of the open access databases (combined and selectivity-based rankings), and local radiality outperforms on the set of targets reported in at least two open access databases. In case of PD, prioritization based on the module triad framework (combined and selectivity-based rankings) outperforms other algorithms on all three sets of the approved targets. We also note that the prioritization methods based purely on genetic associations with PD (RWR and node2vec) or purely on the transcriptomic differences between diseased and control samples (local radiality with respect to the Response module) perform consistently poorly on all three target sets in case of PD, while selectivity to the Treatment module ranks the approved targets higher, suggesting that currently approved targets for PD are mainly functionally similar to the Treatment module genes, and not topologically related to the PD-associated genes. In case of AD, we observe mixed results in case of Citeline-derived approved targets, where the combined module triad ranking performs best in terms of AUC and Precision/Recall@500, but RWR performs best in terms of AUPR. In case of the targets from the open access databases (both versions), the ranking based on the module triad outperforms other approaches on all performance metrics. Overall, our results suggest that the proposed target prioritization may be beneficial not only for identification of novel therapeutic targets in UC, but also in other complex diseases.

**Table 2:** Performance of the proposed module triad method and other network-based target prioritization methods on recovery of known approved targets in psoriasis, Parkinson’s disease and Alzheimer’s disease.

	Module triad prioritization			Reference prioritization methods		
Performance metric	Combined (sel. & prox.)	Selectivity to TM	Proximity to GM	Local radiality	Random walk	node2vec
**Psoriasis**						
**Citeline,** ${\textbf{31}}$ **approved targets**						
AUC	$\mathbf {0.73}$	0.71	0.65	0.70	0.72	0.48
AUPR	0.0039	0.0038	0.0032	0.0049	$\mathbf {0.0166}$	0.0068
Precision@500	0.002	0.006	0.004	0.004	$\mathbf {0.010}$	0.006
Recall@500	0.0322	0.0968	0.0645	0.0645	$\mathbf {0.1613}$	0.0968
**Open access databases (union),** ${\textbf{99}}$ **approved targets**						
AUC	0.72	$\mathbf {0.73}$	0.63	0.63	0.62	0.48
AUPR	$\mathbf {0.0143}$	0.0139	0.0090	0.0114	0.0132	0.0078
Precision@500	$\mathbf {0.024}$	0.020	0.014	0.012	0.022	0.014
Recall@500	$\mathbf {0.1212}$	0.1010	0.0707	0.0606	0.1111	0.0707
**Open access databases (at least two),** ${\textbf{25}}$ **approved targets**						
AUC	0.77	0.75	0.69	$\mathbf {0.79}$	0.73	0.40
AUPR	0.0041	0.0036	0.0031	$\mathbf {0.0134}$	0.0080	0.0016
Precision@500	0.004	0.004	0.004	0.008	$\mathbf {0.012}$	0.002
Recall@500	0.08	0.08	0.08	0.16	$\mathbf {0.24}$	0.04
$$\underline{\varvec{Parkinson's disease}}$$						
**Citeline, ** ${\textbf{13}}$ **approved targets**						
AUC	0.79	$\mathbf {0.82}$	0.65	0.55	0.59	0.51
AUPR	$\mathbf {0.0047}$	0.0046	0.0023	0.0009	0.0009	0.0007
Precision@500	$\mathbf {0.008}$	0.006	0.004	0.002	0.0	0.0
Recall@500	$\mathbf {0.3077}$	0.2308	0.1538	0.0769	0.0	0.0
**Open access databases (union),** ${\textbf{65}}$ **approved targets**						
AUC	$\mathbf {0.79}$	0.78	0.68	0.46	0.50	0.51
AUPR	$\mathbf {0.0185}$	0.0167	0.0115	0.0031	0.0035	0.0033
Precision@500	$\mathbf {0.030}$	$\mathbf {0.030}$	0.018	0.006	0.002	0.0
Recall@500	$\mathbf {0.2308}$	$\mathbf {0.2308}$	0.1385	0.0462	0.0154	0.0
**Open access databases (at least two),** ${\textbf{34}}$ **approved targets**						
AUC	0.85	$\mathbf {0.86}$	0.69	0.46	0.49	0.49
AUPR	$\mathbf {0.0138}$	0.0128	0.0074	0.0020	0.0020	0.0017
Precision@500	$\mathbf {0.020}$	$\mathbf {0.020}$	0.010	0.006	0.002	0.0
Recall@500	$\mathbf {0.2941}$	$\mathbf {0.2941}$	0.1471	0.0882	0.0294	0.0
$$\underline{\varvec{Alzheimer's disease}}$$						
**Citeline,** $\textbf{9}$ **approved targets**						
AUC	0.84	$\mathbf {0.85}$	0.69	0.61	0.59	0.36
AUPR	0.0048	0.0081	0.0015	0.0068	$\mathbf {0.0376}$	0.0031
Precision@500	$\mathbf {0.006}$	0.004	0.0	0.002	0.002	0.002
Recall@500	$\mathbf {0.3333}$	0.2222	0.0	0.1111	0.1111	0.1111
**Open access databases (union), **${\textbf{78}}$ **approved targets**						
AUC	$\mathbf {0.72}$	0.65	0.69	0.53	0.50	0.46
AUPR	0.0125	0.0081	$\mathbf {0.0150}$	0.0059	0.0104	0.0042
Precision@500	$\mathbf {0.020}$	0.008	0.018	0.008	0.010	0.004
Recall@500	$\mathbf {0.1282}$	0.0513	0.1154	0.0513	0.0641	0.0256
**Open access databases (at least two),** ${\textbf{36}}$ **approved targets**						
AUC	$\mathbf {0.67}$	0.62	0.63	0.49	0.48	0.47
AUPR	$\mathbf {0.0049}$	0.0044	0.0037	0.0020	0.0018	0.0018
Precision@500	$\mathbf {0.008}$	0.006	0.004	0.002	0.002	0.0
Recall@500	$\mathbf {0.1111}$	0.0833	0.0556	0.0278	0.0278	0.0

Known approved targets are derived from Citeline database, as well as the three open access databases. Best performing methods are highlighted in bold.

## Discussion

We have presented a network-based framework for prioritizing protein targets as novel therapies for complex diseases, using UC as a showcase. The module triad framework is the first attempt at capturing both formation and successful treatment of disease at the network level assuming that the mechanism behind complex disease formation and treatment can be captured by the interplay between the three network modules of genetic predisposition, transcriptional changes and protein targets of drugs in the HI. In the presented framework, we assume that formation of the disease phenotype is predetermined by the genetic mutations in a collection of genes that are localized in the HI region called the Genotype module. These genetic alterations within the Genotype module manifested in gene expression changes in patients with active UC. By tracking the genes whose expression levels changed significantly in the patients that achieved low disease activity upon TNFi therapy, we derive a collection of genes that need to be transcriptionally altered in order to achieve a positive response to the treatment. These genes occupy a localized region of the HI termed the Response module. Next, we identify proteins targeting which results in a similar transcriptional perturbation profile as achieved upon successful TNFi therapy. We do so by scanning the experimental data of the small molecule compounds perturbing human cells and matching the response profiles after compound perturbation with the profile achieved upon successful treatment. The collection of compound targets that achieve the desired downstream change of gene expression also occupies a localized region in the HI and is called the Treatment module. While the identified compounds matching the desired transcriptomic downstream effect may seem different (see Supplementary Table S4 for their known mechanisms of action), their targets belong to a localized region of the HI, reflecting common underlying biology behind treatment of UC, and suggesting that other protein targets that are functionally similar to the Treatment module nodes are promising targets for UC treatment. By ranking the HI nodes based on their proximity to the Genotype module and selectivity to the Treatment module, we prioritize the HI proteins that are simultaneously topologically relevant to the genes associated with formation of UC phenotype, and functionally similar to proteins that have desirable treatment downstream effect when being targeted.

Proximity used for quantifying topological relevance of targets to Genotype module was previously shown to offer an unbiased measure of therapeutic effects across various drugs and diseases, and for distinguishing palliative from effective treatments^19^. Drugs whose targets are proximal to genes associated with a disease are more likely to be effective than more distant drugs^19^. Here we have used DSD—a metric based on similarity between network random walks—as a proxy for measuring similarity between downstream effects resulting from perturbing a given pair of nodes in the HI. Previously, random walks were successfully used to assess perturbation patterns resulting from elementary mutations in genes related to cancer (single-nucleotide variations and insertion/deletion mutations)^73^. Following the same idea, here we assumed that two nodes having similar downstream perturbation effect on the rest of the network should also have highly similar random walk patterns, measured by DSD metric (see "Methods"), i.e., a pair of nodes with a small DSD corresponds to the nodes with similar downstream perturbation effects. We have demonstrated that DSD is indeed reflective of similarities between therapeutic effects of different targets by recovering known approved targets for six complex diseases, including UC, based on the DSD.

We also note that the target prioritization validation strategy employed here treats all known approved targets as a positive class, while all other targets are considered to belong to the negative class. Rigorously, however, one should treat the rest of the targets as the “unlabeled” class, i.e., containing both positive and negative samples. This approach is called Positive Unlabeled (PU) learning^74^. Despite that, in order to properly evaluate performance of the ranking methods, one should also make additional assumptions on the distribution of the observed positive class examples^74^, and it is not clear whether these assumptions hold in our case. Moreover, as our and other considered baseline approaches are unsupervised and do not rely on learning from the observed positive examples, we can only observe an effect from considering the PU ranking problem in the evaluation metrics. As we tested our method on multiple drug databases and observed gain in performance for our method in the majority of the considered cases, we believe that the evaluation results should hold in PU settings as well. Similarly, approved targets that are widely used in clinic and considered here as the positive class may not necessarily represent a gold standard of targets for validation, as many of them may have low treatment response rate for some cohorts of patients.

The module triad framework utilizes knowledge about the treatment dynamics of patients with active UC that achieved low disease activity upon TNFi therapy. However, patients that do not demonstrate sufficient response to TNFi therapy present a large fraction of diseased population^4^, and may potentially suffer from UC subtype that is different in its underlying biology or disrupts normal cellular processes more severely than in responders (see Supplementary Note S1.3). While novel targets identified using the proposed framework may help finding alternative therapies suitable for TNFi non-responders, understanding of exact biological mechanism behind insufficient response to TNFi therapies is still required for more precise UC treatment recommendations.

In conclusion, the module triad framework that utilizes patients genomic and transcriptomic data offers a holistic network-based view on the formation and treatment dynamics of complex diseases, and provides an unbiased approach to novel target identification. As we have demonstrated for psoriasis, PD and AD use cases, this framework can be generalized to any complex disease with available gene-disease associations data, transcriptomic data of patients before and after treatment, and perturbation experiments in an appropriate cell line. Besides target prioritization, this framework suggests repurposing opportunities based on the targets belonging to the Treatment module. The module triad approach may be enhanced by considering available perturbation experiments such as single-gene overexpression and knockdown, including information about agonist or antagonist action of drugs on their targets, rectifying the Response module by using transcriptomic data of patients undergoing treatment with drugs having alternative mechanisms of action, or by further refining the list of prioritized targets considering their toxicity and druggability.

## Methods

### Human Interactome

The HI map of experimentally derived protein-protein interactions is assembled from 21 public databases in the same way as previously described in^17,75^. The HI used in this paper is assembled using database versions as of March 2021. The databases contain multiple types of protein-protein interactions derived from high-throughput yeast-two hybrid experiments, three-dimensional protein structures, literature curation, affinity purification followed by mass spectrometry, kinase/substrate interactions, signaling and regulatory interactions. We use the largest connected component (LCC) of the resulting undirected network constructed from the pairs of interacting proteins which we refer to as the HI.

### Construction of the UC Genotype module

Genes associated with UC are identified as indicated by the (1) GWAS catalog^34^; (2) ClinVar database^35^, specifically, genes that are indicated as “pathogenic”, “likely pathogenic”, and with “conflicting interpretations” of pathogenicity; and (3) MalaCards database^36^. The genes are collected from the databases as of September 2021. We retain all the genes that are mentioned in at least one of the three databases, and further filter out the genes that are not part of the HI network. We use the remaining genes to construct a subnetwork, and extract the largest connected component (LCC) of it.

We assess significance of the LCC size by randomly sampling subnetworks with the degree sequence as in the original subnetwork. By repeatedly sampling 10, 000 subnetworks, we find the empirical distribution of the LCC size of randomly sampled subnetworks with its mean $\mu _{LCC}$ and standard deviation $\sigma _{LCC}$. We define the LCC *Z*-score as:

1 $$\begin{aligned} Z_{LCC} = \frac{S_{LCC} - \mu _{LCC}}{\sigma _{LCC}}, \end{aligned}$$

where $S_{LCC}$ is the LCC size of the original subnetwork. We also define the empirical *p*-value for the observed $S_{LCC}$ as the fraction of the randomly sampled subnetworks that had their LCC size exceeding $S_{LCC}$.

### Gene expression data processing for active UC cases and normal controls

We collect tissue mucosal samples from normal controls and patients with moderately to severely active UC from Gene Expression Omnibus (GEO)^76^, see Supplementary Table S1 for details. Three studies reported patient response statuses after treatment, where responses are determined by endoscopic and histologic findings or Mayo scores, see Supplementary Table S2 for details on the response definition. We obtain normalized data within each study from GeneVestigator^77^. We integrate the expression data from 6 infliximab studies together. Batch effects among different studies are corrected using ComBat^78^. Some studies include baseline samples and samples collected at follow-up visits. To avoid underestimating variance introduced by analysis of longitudinal correlated samples, we apply ComBat to only baseline samples to derive correction factors for individual studies, treating response and health status as covariates. The correction factors are implemented on baseline and follow-up visit samples.

### Clustering and differential gene expression analysis

To reduce dimensionality of the gene expression data, we first select a subset of gene features that are significantly differentially expressed between normal controls and UC active samples. We extract genes with fold change (FC) of $FC > 2.5$ and adjusted *p*-value (Benjamini-Hochberg correction^79^) of $p_{adj.} < 0.05$. For clustering analysis, we embed gene expression vectors of the identified differentially expressed genes into 8-dimensional space using UMAP^44^.

When comparing the pre- and post-treatment gene expression profiles of the active UC patients, we use $FC > 1.8$ and $p_{adj.} < 0.05$ thresholds to identify differentially expressed genes. The differentially expressed genes with negative log-fold change are considered significantly down-regulated, while genes with positive log-fold change are considered significantly up-regulated. For more details on the paired analysis of differentialy expressed genes, see Supplementary Note S1.1.

### Construction of the UC Response module

To identify genes indicative of response to TNFi therapy, we first extract the genes that are significantly differentially expressed in responders to infliximab and golimumab comparing their gene expression profiles before and after treatment as described above. We obtain the two RBA gene sets from infliximab- and golimumab-based studies (see Supplementary Note S1.1 for details), and use a union of these two sets to account for possible drug-specific gene expression changes. We then construct a subnetwork based on the obtained merged RBA gene set and the HI. We identify the LCC of the resulting subnetwork as the UC Response module, and assess significance of its size analogously to the Genotype module.

### Analysis of LINCS L1000 perturbation profiles

We assess the concordance between the differential gene expression profile upon perturbation of HT29 cells using various compounds and the genes belonging to the Response module split into up- and down-regulated subsets using Weighted Connectivity Score (WTCS)^23^. WTCS measures the enrichment score^80^, *ES*, of ranked lists of genes with a given pair of up- and down-regulated gene sets, that are referred to here as up- and down-query. WTCS combines the ES for up-query ($ES_{up}$) and down-query ($ES_{down}$) into a single score. A positive WTCS indicates that a perturbation resulted in a gene expression change that aligns with the Response module query set, i.e., up-query genes are also mainly up-regulated in a given perturbation, and down-query genes are mainly down-regulated in a given perturbation. Conversely, a negative WTCS indicated that down-query genes are up-regulated in a given experiment, and up-query genes are down-regulated. As we are interested in reverting expression patterns of the Response module genes, we look for experiments with negative WTCS. Below is the brief outline of the procedure used to compute this score and to assess its statistical significance.

LINCS L1000 Level 5 data stores differential gene expression profiles in terms of gene-specific *Z*-scores indicating changes in expression levels of genes with respect to controls. Large positive *Z*-score indicates that a gene is significantly up-regulated upon perturbation, while large negative *Z*-score indicates that a gene is significantly down-regulated upon perturbation. Genes for which differential expression patterns are inferred with high fidelity belong to the set of *Best INferred Genes* (BING)^23^ and are used for WTCS computation. Up-regulated and down-regulated genes observed in the Response module that are also part of the BING set are denoted here as $s_{up}$ and $s_{down}$, respectively. For each sets, we calculate enrichment scores $ES_{up}$, $ES_{down}$, and WTCS is a combination of these two scores:

2 $$\begin{aligned} WTCS = {\left\{ \begin{array}{ll} \frac{1}{2} \left( ES_{up} - ES_{down} \right) , &{} \text {if} {{\,\textrm{sign}\,}}{(ES_{up})} \ne {{\,\textrm{sign}\,}}{(ES_{down})} \\ 0, &{} \text {otherwise.} \end{array}\right. } \end{aligned}$$

To assess the significance of the enrichment scores, we sample genes sets of sizes $\left| s_{up}\right|$, $\left| s_{down}\right|$ uniformly from BING genes. By repeating the sampling procedure 1, 000 times, we obtain empirical distributions of up- and down-enrichment scores from random samples, $\rho _{up}\left( ES\right)$, $\rho _{down}\left( ES\right)$. We then compare the obtained distributions to the observed $ES_{up}$ and $ES_{down}$: if the observed $ES_{up}$ is positive, the fraction of random samples which has greater or equal enrichment scores is selected as the *p*-value $p_{up}$, and if it is negative, the fraction of random samples which has smaller or equal enrichment scores is selected as the *p*-value $p_{up}$. The $p_{down}$ is computed in a similar fashion. As a result, we obtain WTCS, $p_{up}$, and $p_{down}$ for each perturbation experiment and use them for filtering the relevant perturbations.

### Construction of UC Treatment module

Using LINCS L1000 data, we identify compounds that are able to revert the expression patterns observed in the Response module nodes. We extract relevant experiments using $WTCS < 0$ and $p_{up} < 0.05$, $p_{down} < 0.05$ filters described above. The protein targets of the compounds remained after the filtering are identified using DrugBank and Repurposing Hub databases. We then map the resulting set of protein targets on the HI, and construct a subnetwork based on it analogously to the construction of the Response and Genotype modules. Treatment module is the LCC of this subnetwork.

### Diffusion state distance

Diffusion state distance (DSD) is a metric defined on network nodes originally designed to predict proteins’ functions in protein interaction networks^26^. DSD captures similarities between network’s final states when random walkers start from two different nodes. To define the DSD, we first define $He(v_i, v_j)$ – an expected number of times a random walk (RW) starting at node $v_i$ and proceeding for *k* steps will end up at node $v_j$. Next, for node $v_i$, we define a vector

3 $$\begin{aligned} He(v_i) = \{ He(v_i, v_1), \ldots , He(v_i, v_n) \}. \end{aligned}$$

Then the DSD between nodes $v_i$ and $v_j$ is defined as

4 $$\begin{aligned} DSD(v_i, v_j) = || He(v_i) - He(v_j) ||_1, \end{aligned}$$

where $||\ldots ||_1$ denotes the $L_1$ norm. For any fixed *k*, DSD is a metric and it converges as $k\rightarrow \infty$^26^.

### DSD as a measure of therapeutic similarity between targeted proteins

To quantify relevance of DSD as a measure of therapeutic effect similarity between proteins, we analyze a set of complex diseases and their approved targets. First, for each of the known approved targets for a given disease, we compute DSDs between that target and the rest of the nodes in the HI. Second, we rank the rest of the nodes based on the DSD to a known target, and based on that ranking, we construct a receiver operator characteristic (ROC) and Precision-Recall (PR) curves corresponding to the recovery of the rest of the approved targets for a given disease. By iterating over all known approved targets, a set of individual ROC curves is obtained for each of complex diseases. We use interpolation to average the individual curves and to obtain the mean ROC/PR curve, and compute the area under it, quantifying the likelihood of finding approved targets given only the knowledge about a single approved target and its DSD to the rest of the network nodes.

### Proximity to UC Genotype module

Computing proximity of a node to the Genotype module consists of the two steps. First, we compute the average shortest path length ${\bar{d}}$ from a given node to the nodes of the Genotype module. Second, we assess the statistical significance of the closeness of the node to the Genotype module by comparing the average shortest path length to the Genotype module to the average shortest path distance to randomized network modules of the same size. Specifically, we sample connected modules of the same size as the Genotype module (see below for sampling details) 500 times, and construct an empirical distribution of the average shortest path distances to the randomized modules, with $\mu _p$ being the mean, and $\sigma _p$ being the standard deviation of this distribution. Finally, proximity of the node is defined as the *Z*-score of the average shortest path distance from the node to the Genotype module with respect to this distribution:

5 $$\begin{aligned} proximity = \frac{{\bar{d}} - \mu _p}{\sigma _p}. \end{aligned}$$

### Selectivity to UC Treatment module

Computing selectivity of a node to the Treatment module is similar to computation of proximity. First, we compute the average DSD (${\overline{DSD}}$) of a node with respect to the nodes of the Treatment module. Second, we assess the statistical significance of the observed DSD by sampling 500 randomized network modules of the same size as the Treatment module, analogously to the proximity calculation. However, instead of the average shortest path distance, we compute the average DSD of the node to each randomized module, and construct an empirical distribution of the average DSDs to the randomized modules, with $\mu _s$ being the mean and $\sigma _s$ being the standard deviation of this distribution. We define selectivity as:

6 $$\begin{aligned} selectivity = \frac{{\overline{DSD}} - \mu _s}{\sigma _s}. \end{aligned}$$

### Network module randomization

Both proximity and selectivity computations require sampling of randomized modules on the HI. As by construction both Genotype and Treatment modules are connected subnetworks, we have to sample connected subnetworks uniformly from the fixed HI network in order to avoid any possible biases of the average shortest path length or DSD with respect to the subnetwork connectedness. To this end, we employ Neighbor Reservoir Sampling (NRS) algorithm^81^ to sample connected fixed-size subnetworks uniformly.

### Node ranking based on proximity and selectivity

Given the Genotype and Treatment modules, we compute proximity and selectivity scores of all nodes in the HI, and derive their corresponding ranks, $r_p$ and $r_s$, respectively. To obtain a single combined rank *r* for each node, we used the rank product defined as

7 $$\begin{aligned} r = \sqrt{r_p \cdot r_s}. \end{aligned}$$

### Local radiality with respect to the Response module

LR is a measure based both on network and gene expression data, and have shown high performance in recovering known drug targets^21^. LR is similar to the proposed module triad prioritization in that it employs both topological and gene expression data to prioritize targets. The main difference is that Local radiality assumes that HI nodes affected by perturbation of a target (downstream nodes) should be in the network vicinity of this target. In our settings, this means that targets should be prioritized based on their Local radiality with respect to the Response module nodes that reflect the desired downstream effect.

We measure Local radiliaty of node *i* with respect to the Response module using the following equation:

8 $$\begin{aligned} LR_i = \frac{\sum _{g \in RM} spl(i, g, G)}{|RM|}, \end{aligned}$$

where *RM* is the set of the Response module nodes, *G* is the Human Interactome network, *spl*(*i*, *g*, *G*) is the function measuring the length of the shortest path from node *i* to node *g*.

### Random walk with restart (RWR)

RWR allows to compute probabilities of ending up at a particular HI node for a random walk that starts at a predefined set of seed nodes. At each step of the random walk, the random walker either jumps to one of the current node’s neighbors or restarts the walk with a fixed probability controlled by the parameter $\alpha _{RWR} \in (0, 1)$. In this setting, RWR algorithm is also known as the Personalized PageRank^82^. The RWR algorithm has shown good performance for many gene prioritization tasks^83,84^, and is often used as a baseline reference algorithm^48^.

We use Personalized PageRank implementation of RWR provided in the NetworkX library. We use all genes associated to a disease that can be mapped to the HI as the seed nodes with the same personalization value of 1/|*S*|, where *S* is the set of seed nodes. We keep the default restart probability parameter $\alpha _{RWR} = 0.85$, and set the error tolerance parameter *t* for eigenvalue solver to $t = 1 \cdot 10^{-8}$. As an output of this algorithm, we obtain a list of RWR scores $p_{i}$ for each node *i*. We use these scores to rank all nodes in the score-descending order, assuming that the higher scores correspond to the nodes that are more likely to be good targets for disease treatment.

### Node2vec embedding

Node2vec^49^ is a network embedding method that represents network nodes as points in low-dimensional Euclidean space. Intuitively, node2vec generates vector representation for nodes in such a way that nodes coming from similar network neighborhood tend to have similar vector representations, i.e., are close in the embedding space. Using this measure of similarity between nodes, one can prioritize HI nodes with respect to a predefined seed node set. Previously, node2vec was shown to perform well in recovering gene-disease associations for Parkinson’s disease^85^.

We use a Python implementation of node2vec embedding algorithm provided in the original paper^49^. We use the default settings of the algorithm provided in the package. As an output of the algorithm, we obtain 128-dimensional vector representations $\textbf{x}_i$ for each node in the HI. We then use the cosine similarity score proposed in^48^ to assess similarity between any given node *i* and the set of disease-associated genes *S*:

9 $$\begin{aligned} \text {n2v}_i = max \left\{ \frac{\textbf{x}_i \cdot \textbf{x}_j}{\left\| \textbf{x}_i \right\| _2 \left\| \textbf{x}_j \right\| }_2 , j \in S \right\} , \end{aligned}$$

where $\left\| \ldots \right\| _2$ denotes $L_2$-norm. We note that nodes belonging to the set *S* are assigned $\text {n2v}$ score of 1. We rank all nodes in the score-descending order, similar to the RWR ranking.

### UC approved targets (Citeline)

For validation of the proposed target prioritization framework, we compile a list of targets that are approved for UC treatment. To do this, we first retrieve a list of all drugs with a status of launched or in development for UC using the Citeline database^45^ as of February 2022. All drugs that are launched for UC are considered as approved drugs. We additionally consider drugs that are being tested for UC in clinical trials (Phase I, II, and III) and preclinical trials to compare their combined rankings to those of the approved drugs. For each drug, we extract its known targets from Citeline, Repurposing Hub, and DrugBank databases. Since a target may be mapped to several drugs, we assign the highest reached status to a target based on the statuses of the drugs it is mapped to. For example, if a target is mapped to the two drugs, one of which is in Phase II clinical trials, and one is in preclinical trials, the target is labelled as the clinical trials target. Moreover, to avoid drugs that may have potentially many off targets due to high drug promiscuity^86^, we filter out the two drugs (sulfasalazine and mesalazine) that have more than 4 targets (see Supplementary Figure S3 for details). Besides these two drugs, all other drugs being developed for UC treatment have 4 or less targets simultaneously. Additionally, we filter out tetracosactide due to ambiguous indications for UC. Targets for psoriasis, Parkinson’s disease and Alzheimer’s disease are extracted in similar way, but without filtering by the number of reported targets per drug.

### UC approved targets (open access databases)

We use the following three open access databases to extract approved targets for UC treatment: Repurposing Hub, DrugBank, and Therapeutic Target Database. In Repurposing Hub, we search for all drugs with the “launched” status and “ulcerative colitis” listed in the indications, and extract their known targets. In DrugBank, we search for drugs by indication of “ulcerative colitis” which also returns drugs indicated for the subtypes of UC, e.g., “moderate to severe ulcerative colitis” or “mild ulcerative colitis”. We manually curate all drugs indicated for either of the UC forms, and extract their known targets. In Therapeutic Target Database, we search targets by disease using “ulcerative colitis” as the search term, and mapping the yielded protein targets back to their gene symbols if applicable. Targets for psoriasis, Parkinson’s disease and Alzheimer’s disease are extracted in similar way.

## Supplementary Information

Supplementary Information.

## Data Availability

Data used to produce the figures are provided with the manuscript. Gene expression data of UC patients and healthy controls are based on publicly available datasets listed in Supplementary Table S1, and normalized by Genevestigator^77^. Normalized datasets may be obtained from Genevestigator. Code implementation of the method is available from the authors upon reasonable request.
