# Supplementary material for: The module triad: a novel network biology approach to utilize patients’ multi-omics data for target discovery in ulcerative colitis
Source: Sci Rep. 2022 Dec 15;12:21685. doi: 10.1038/s41598-022-26276-x (PMC9755270; doi:10.1038/s41598-022-26276-x)
Supplement: Supplementary file 1 — Supplementary Information. [file 41598_2022_26276_MOESM1_ESM.pdf]

S1 Supplementary information: The Module Triad: a novel network biology approach to utilize patients' multi-omics data for target discovery in ulcerative colitis

Ivan Voitalov[a], Lixia Zhang[a], Casey Kilpatrick[a], Johanna B. Withers[a], Alif Saleh[a], Viatcheslav R. Akmaev[a], Susan Dina Ghiassian[a]

[a]*Scipher Medicine Corporation, 221 Crescent St Suite 103A, Waltham, MA 02453*

S1.1 Supplementary note: differential gene expression analysis of responders and non-responders to TNFi therapy

To assess if responders and non-responders to TNFi therapies can be stratified based on gene expression profiles before treatment, we perform differential gene expression analysis using their full gene expression profiles. We do not find any significant differences at the fold change (FC) of $FC = 1.8$ and adjusted p-value (Benjamini-Hochberg correction) of $p < 0.05$. Therefore, we demonstrate that there are no evident differences between responders' and non-responders' before treatment neither in the UMAP embedding space, nor in the actual full gene expression profile space.

Motivated by the fact that before treatment UC active patients' gene expression profiles are not enough to distinguish responders from non-responders, we additionally consider normal tissue controls as a comparison reference to derive more evident difference in the gene expression profiles between responders and non-responders. We construct the following four sets of differentially expressed genes, comparing different groups of patients and normal controls (see Figure S1 for illustration of the sets):

1. Responders-before-after set (RBA): differentially expressed genes in responders between before- and after-treatment;
2. Non-responders-before-after set (NRBA): differentially expressed genes in non-responders between before- and after-treatment;
3. Responders set (R): differentially expressed genes between baseline responders and normal controls;
4. Non-responders set (NR): differentially expressed genes between baseline non-responders and normal controls.

Each of these paired states are measured separately in infliximab- and golimumab-based studies.

First, we observe that non-responders do not show significant changes in gene expression profiles upon treatment, thus NRBA does not contain any significantly differentially expressed genes. Second, we observe that R, NR, and RBA sets are highly concordant and have significant intersection size both for infliximab and golimumab studies, Figure S1, panel (b). Pairwise hypergeometric test yields $p = 9 \cdot 10^{-910}$ and $5 \cdot 10^{-1249}$ for the intersection between NR and R sets, $p = 4 \cdot 10^{-64}$ and $8 \cdot 10^{-91}$ for intersection between NR and RBA sets, $p = 2 \cdot 10^{-226}$ and $1 \cdot 10^{-103}$ for intersection of R and RBA sets in infliximab and golimumab studies, respectively.

Moreover, most RBA genes are differentially expressed in baseline responder samples relative to normal controls, indicating that treatment with a TNFi results in reversion of the expression of a small subset of R genes. On the contrary, despite the significant fraction of RBA genes is contained within the NR set, these genes are not significantly altered in non-responders after treatment with TNFi.

The RBA gene sets are almost exclusively comprised of genes contained within the R and NR sets. Moreover, as suggested by UMAP plots (Figure 2), the gene expression profiles of responders after treatment is closer to that of normal controls, while non-responders after treatment remain close to their initial pre-treatment position in the UMAP space. This suggests that to achieve low disease activity in responders, it is sufficient for TNFi treatment to revert the expression profile of a subset of the differentially expressed genes constituting the RBA set.

S1.2 Supplementary note: module triad construction for psoriasis, Parkinson's disease (PD) and Alzheimer's disease (AD)

To check how our target prioritization pipeline generalizes to other complex diseases, we repeat prioritization validation using known approved targets for three complex diseases – psoriasis, PD and AD. These diseases are picked from the 6 diseases we have used previously to demonstrate the utility of the DSD metric (see Supplementary Figure S5). As the focus of this paper is ulcerative colitis, we check performance of the module triad method only on the diseases that have readily available data for the construction of the three modules. For Response modules, we use Harmonizome database [1] to extract differentially expressed genes associated to the diseases, as extracting and normalizing relevant datasets from various public databases separately for each disease would be a laborious task.

Therefore, in our analysis we omit multiple sclerosis (MS), as Harmonizome lists two datasets related to MS, one based on the B cell lymphocyte samples for which we do not have any reasonable cell line model in LINCS database, and another based on the spinal cord samples derived from mice. We also omit considering generalizability of the framework in rheumatoid arthritis (RA) as we do not have any cell line in LINCS database that would be related to the synovial tissue affected in RA patients. For the rest of the complex diseases, we describe the construction of the corresponding module triad below.

Psoriasis Genotype module. We build psoriasis Genotype module by selecting "elite" genetic associations from Malacards, "pathogenic", "likely pathogenic", and with "conflicting interpretations of pathogenecity" genetic associations from ClinVar, and all genetic associations from GWAS Catalog. Overall, we find 121 genetic associations reported in either of the three databases, and 96 of them can be mapped to one of the HI nodes. We observe that only 27 of 96 genes form the largest connected component (LCC) which is barely significant under degree-preserving randomization (Z-score $= 1.76$, $p = 0.04$, degree-preserving randomization). As we observe that the majority of genetic associations are discarded from the LCC, we choose to include all 96 subgraph nodes in the psoriasis Genotype module. For consistency, we measure the proximity to Genotype module with respect to random subgraphs of the same size, without considering their connectivity, as the original observed subgraph is not fully connected.

PD Genotype module. We build PD Genotype module by selecting "elite" genetic associations from Malacards, "pathogenic", "likely pathogenic", and with "conflicting interpretations of pathogenecity" genetic associations from ClinVar that specify the term "Parkinson disease" in the associated condition field, and all genetic associations from GWAS Catalog. Overall, we find 137 genetic associations reported in either of the three databases, and 109 of them can be mapped to one of the HI nodes. We observe that only 24 of 109 genes form the largest connected component (LCC) which is barely significant under degree-preserving randomization (Z-score $= 1.77$, $p = 0.04$, degree-preserving randomization). As we observe that the majority of genetic associations are discarded from the LCC, we choose to include all 109 subgraph nodes in the PD Genotype module. Similar to the case of psoriasis, we measure the proximity to the PD Genotype module with respect to random subgraphs of the same size, without considering their connectivity.

AD Genotype module. We build AD Genotype module by selecting "elite" genetic associations from Malacards, "pathogenic", "likely pathogenic", and with "conflicting interpretations of pathogenecity" genetic associations from ClinVar that specify the term "Alzheimer disease" in the associated condition field, and all genetic associations from GWAS Catalog. Overall, we find 209 genetic associations reported in either of the three databases, and 163 of them can be mapped to one of the HI nodes. We observe that 61 of 163 genes form the largest connected component (LCC) which is significant under degree-preserving randomization (Z-score $= 1.77$, $p = 0.02$, degree-preserving randomization). As we the LCC covers about one third of all genetic associations mapped to the HI, we choose to use this LCC as the AD Genotype module. Unlike the cases of psoriasis and PD, we measure the proximity to the AD Genotype module with respect to random *connected* subgraphs of the same size.

Psoriasis Response module. We derive the list of significantly differentially expressed genes for patients with psoriasis with respect to normal controls using Harmonizome database [1]. Harmonizome database lists gene sets differentially expressed in patients diagnosed with a disease with respect to normal controls. As we do not have access to an extensive collection of transcriptomic datasets that compare psoriasis patients before and after successful treatment as we do for the case of UC, we resort to comparing psoriasis patients before treatment and normal controls, assuming that successful treatment of psoriasis imposes transcriptomic changes in patients such that they become similar to normal controls. We find 3 psoriasis datasets listed in Harmonizome (GEO accession numbers GSE13355, GSE14905, GSE6710). Each of the datasets lists 600 significantly differentially expressed genes. We observe that these gene sets are not largely consistent with each other (see Supplementary Figure S10), however, 64 DE genes are reported in two sets simultaneously. Therefore, we extract these consistently observed DE genes and map them on the HI. 52 of 64 are mapped on the HI, and only 4 out of 52 genes form an LCC. The size of the LCC is not significant

(Z-score -1.03, p-value 0.77, degree-preserving randomization), therefore, we use the full subgraph of 52 nodes as the psoriasis Response module.

PD Response module. Similarly to the psoriasis case, we use Harmonizome to extract differentially expressed genes for patients with PD with respect to normal controls. The only dataset related to PD lists DE genes of PD patients in samples collected postmortem from substantia nigra (GEO accession number GSE7621). We find that of 600 DE genes reported by Harmonizome, 537 can be mapped on the HI, and 439 of them form an LCC. However, the size of this LCC is not significant (Z-score 0.67, p-value 0.23, degree-preserving randomization), therefore, we use the full subgraph of 537 nodes as the PD Response module.

AD Response module. Harmonizome contains two datasets related to AD: GSE5281 and GSE1297. The latter is based on the samples collected from hippocampus, while the former is based on the samples from entorhinal cortex. However, upon further inspection of the original GEO entry of the GSE5281 dataset, it appears that it also contains multiple samples collected from hippocampus. However, for GSE5281, Harmonizome only reports DE genes associated with the entorhinal cortex samples. We therefore derive DE genes for both sets from scratch using the GEO2R tool provided at GEO and selecting the samples collected from hippocampus. We find that GEO2R reports no significant DE genes in GSE1297 dataset based on the adjusted p-value, so we use the unadjusted p-value in this dataset. We use a p-value threshold of 0.05 for both datasets, and extract genes that have consistent sign of the log-fold-changes across the two datasets. We extract all genes that pass the significance threshold, and have $|log_2(FC)| > 0.5$ in both datasets. We obtain 341 genes after this filtering, and 335 of them can be mapped on the HI. Moreover, 217 out of 335 genes form an LCC of significant size (Z-score 1.90, p-value 0.02, degree-preserving randomization). We use this LCC as the AD Response module.

Psoriasis Treatment module. We construct the Treatment module of psoriasis following similar procedure as described for UC in the main text. As the psoriasis Response module was constructed by considering samples from the skin tissue, in LINCS database, we consider compound perturbation experiments in A375 cell line (Phase I and II), as it has the largest number of compound experiments among all skin-derived cell lines covered by LINCS. We find a total of 51 compound experiments with known protein targets that pass statistical significance and WTCS score filters. We extract a total of 139 protein targets from these compounds (136 of them are mapped on the HI), and observe that 101 of them form an LCC of significant size (Z-score 5.33, p-value $< 10^{-4}$, degree-preserving randomization). We use this LCC as the psoriasis Treatment module. As the Treatment module is connected, we use connected randomized subgraphs for selectivity calculation, as was done in the case of UC.

PD Treatment module. The PD Response module is constructed using the brain-derived samples, therefore, we consider compound perturbation experiments in NEU cell line from LINCS (Phase I and II) to construct the PD Treatment module. The NEU cells are primary terminally differentiated neuron cells. We do not select SHSY5Y cell line, which is more frequently used as a model for PD [2], for our analysis as there are no compound perturbation experiments in this cell line covered in LINCS. We find a total of 6 compound experiments with known protein targets that pass statistical significance and WTCS score filters. We extract a total of 21 protein targets from these compounds (20 of them are mapped on the HI), and observe that 3 of them form an LCC of insignificant size (Z-score 0.23, p-value 0.12, degree-preserving randomization). We therefore use the full subgraph of 20 nodes as the PD Treatment module. As the Treatment module is unconnected, we use unconnected randomized subgraphs for selectivity calculation.

AD Treatment module. The AD Response module is constructed using the hippocampus-derived samples, therefore, we consider compound perturbation experiments in NEU cell line from LINCS (Phase I and II) to construct the AD Treatment module. We find a total of 26 compound experiments with known protein targets that pass statistical significance and WTCS score filters. We extract a total of 93 protein targets from these compounds (91 of them are mapped on the HI), and observe that 73 of them form an LCC of significant size (Z-score 4.72, p-value $< 10^{-4}$, degree-preserving randomization). We use this LCC as the AD Treatment module. As the Treatment module is connected, we use connected randomized subgraphs for selectivity calculation.

S1.3 Supplementary note: pathway enrichment analysis of differentially expressed genes in responders and non-responders to TNFi therapy

To have a better understanding of the underlying molecular mechanisms of non-response, we perform pathway enrichment analysis on the R and NR sets. For each of the KEGG pathways [3], we determine the fraction of nodes that are part of the R and NR gene sets (Figure S2, see below for details). Of 282 KEGG pathways that include at least one gene from the R and NR sets, 40 pathways are significantly enriched with NR genes (hypergeometric test, $p < 0.05$). The majority of the genes in these pathways are common to the NR and R sets. To identify pathways that are more enriched in NR-exclusive genes, we perform a statistical test based on random sampling to assess the significance of difference between the number of NR-exclusive versus R-exclusive genes within the pathway (see below for details). From the 40 pathways, 28 have significantly more NR-exclusive genes than R-exclusive genes are retained ($p < 0.05$) (Figure S2, panel (c)). Pathways relevant to UC such as "Inflammatory bowel disease", "TNF signaling pathway", "Intestinal immune network for IgA production", "Rheumatoid arthritis", "Cell adhesion molecules", "IL-17 signaling pathway" are significantly more disrupted in non-responders. This observation is supported by pathway enrichment analysis done using Enrichr [4]. The Enrichr analysis reports a nearly identical list of enriched biological pathways between the R and NR gene sets; however, individual pathways tend to have a greater number of genes, p-value and q-values for the NR gene set. The differentially expressed genes unique to non-responders among these pathways include genes involved in cytokine signaling (*IL6, OSM, IL1A, IL1R1, IL11, CXCL8/IL8, IL21R*), receptor mediation (toll-like receptors, *TLR1, TLR2, TLR8*) and signal transduction (Src-like kinases: *HCK, FYN*).

UC-relevant KEGG pathways are more enriched in NR-exclusive genes than that of responders (Figure S2, panel (c)). Unsurprisingly, this includes other inflammatory conditions such as rheumatoid arthritis and diabetes, and likely represents general immune system disfunctions common to these conditions. An estimated 25-35% of patients with an autoimmune disease may develop one or more additional autoimmune disorders [5, 6]. Other enriched pathways highlighted the role of the intestinal microbiome in ulcerative colitis. Genes annotated in the intestinal immune network for IgA production are enriched among non-responders. IgA antibodies are the primary secreted immunoglobulins, and pro-inflammatory bacterial taxa may be more significantly coated with IgA in inflammatory bowel disease patients than healthy controls [7]. Specifically, *Staphylococcus aureus* infection is one enriched bacterial KEGG pathway. Gram positive bacteria such as *S. aureus* induce TNF-α secretion from macrophages, and TNF-α enhances neutrophil-mediated bacterial killing[8]. Perturbation of TNF-α affects the ability of immune system to control an *S. aureus* infection, leading to an elevated risk of infection after TNFi treatment [9]. Innate immunity plays an important role in maintaining intestinal homeostasis, as highlighted by the TLR and NOD-like signaling KEGG pathways. TLR pattern recognition receptors detect conserved structures of microbes, including those of the gut microbiota, and, upon activation, induce inflammatory signaling pathways and regulate antibody-producing B cell responses [10, 11]. TLR2, 4, 8 and 9 are upregulated in the colonic mucosa of patients with active UC relative to quiescent UC or healthy control samples [12]. Cytokine signaling, including the TNF-α and IL-17 pathways, are enriched among non-responders. IL-17 signaling, in addition to being a potent pro-inflammatory cytokine that amplifies TNF-α and IL-16 signaling, induces genes to recruit and activate neutrophils and promotes expression of epithelial barrier genes [13, 14]. Additional disruption of colonic epithelial barrier integrity in non-responders is highlighted through the enrichment of genes in the cell adhesion molecules and fluid shear stress KEGG pathways. Loss of barrier integrity increases the permeability of nutrients, water, bacterial toxins and pathogens across the epithelial barrier [15]. Overall, the pathways that are more significantly enriched suggest that UC disease biology – inflammation, barrier integrity and microbiome disequilibrium – is more broadly disrupted among TNFi non-responders.

To determine if the gene expression profile of non-responders is more severely dysregulated in comparison to that of responders with respect to various pathways, we perform enrichment analysis of signaling pathways from the Kyoto Encyclopedia of Genes and Genomes (KEGG) database. First, pathways that are significantly enriched with non-responders' differentially expressed genes are selected using the significance threshold of $p_{adj.} < 0.05$ (hypergeometric test with Benjamini-Hochberg correction). Second, for each selected pathway, genes that are coming exclusively from the R and NR gene sets are identified. Next, we compute the difference between the number of these R- and NR-exclusive genes, and assess its significance using the random permutation of R- and NR-exclusive labels on the remaining genes. Only the pathways for which there is a significant difference between the number of NR-exclusive and R-exclusive genes are retained ($p_{adj.} < 0.05$, random permutation test with Benjamini-Hochberg correction).

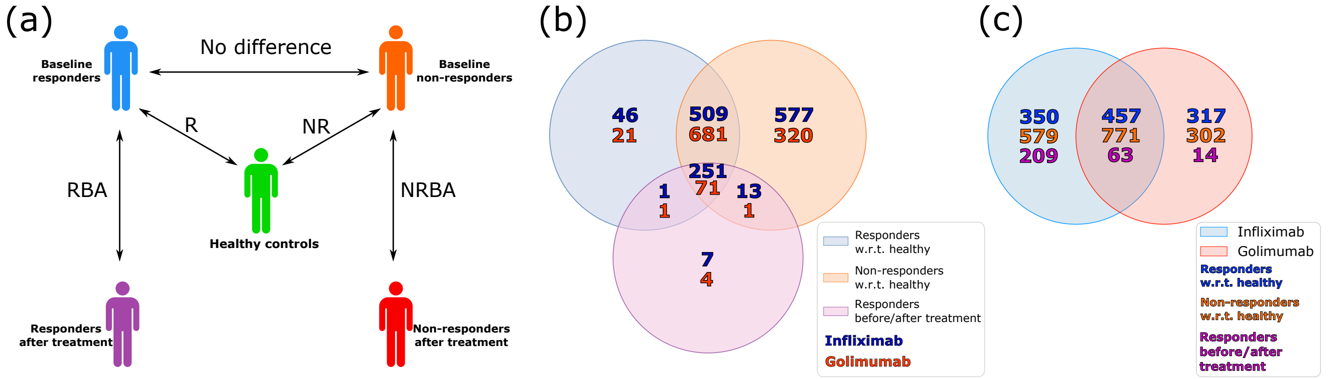

Figure S1: **Overview of the DE analyses performed in this paper. (a)**: schematic illustration of the differential expression gene sets obtained by comparing different pairs of states of responders, non-responders, and normal controls, with the DE genes set names used throughout the paper specified; **(b)**: Venn diagrams for R, NR, and RBA sets in infliximab and golimumab studies; **(c)**: mutual overlaps of R, NR, and RBA sets across the studies.

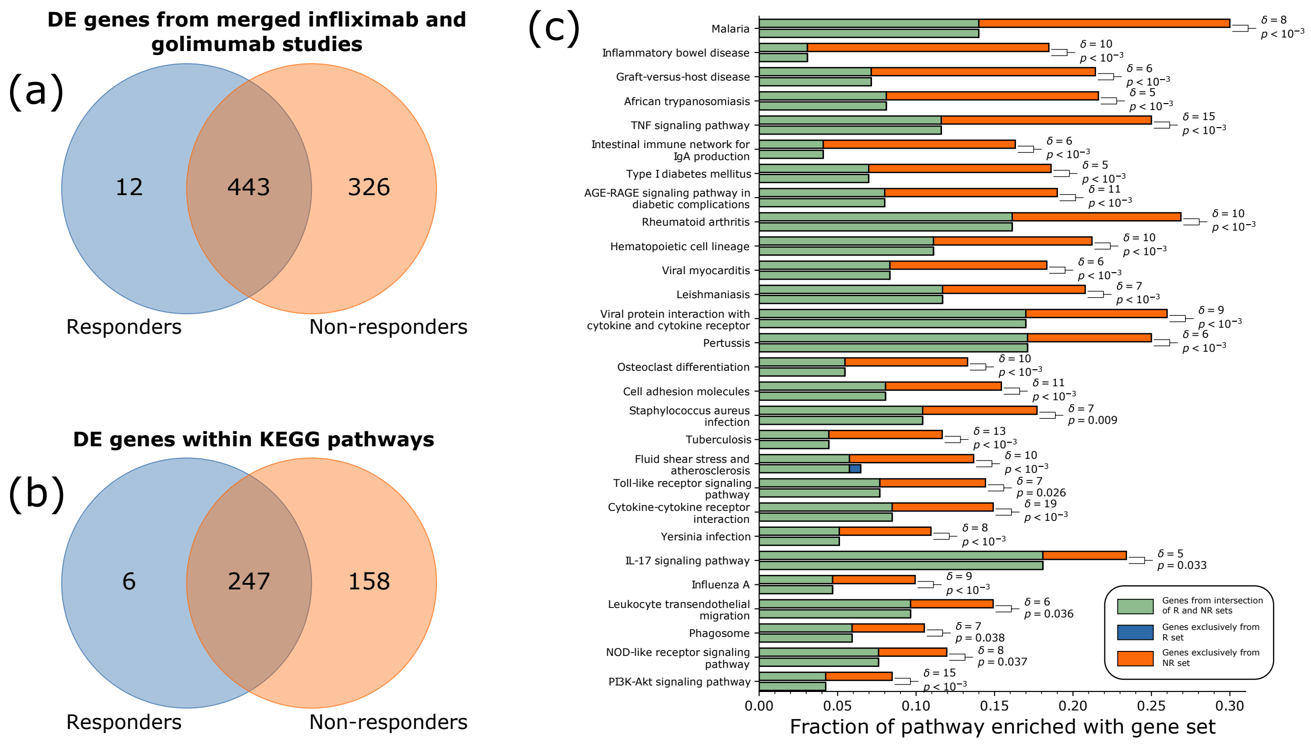

Figure S2: **KEGG pathway enrichment analysis for genes differentially expressed in responders and non-responders at the baseline with respect to healthy controls.** **(a)** Venn diagram for responders' (R) and non-responders' (NR) differentially expressed genes at the baseline with respect to healthy controls after merging the infliximab- and golimumab-based cohorts. **(b)** Venn diagrams for the same gene sets within the KEGG pathways database. **(c)** KEGG pathways significantly enriched with NR gene set that also have significantly more NR-exclusive genes than R-exclusive genes.

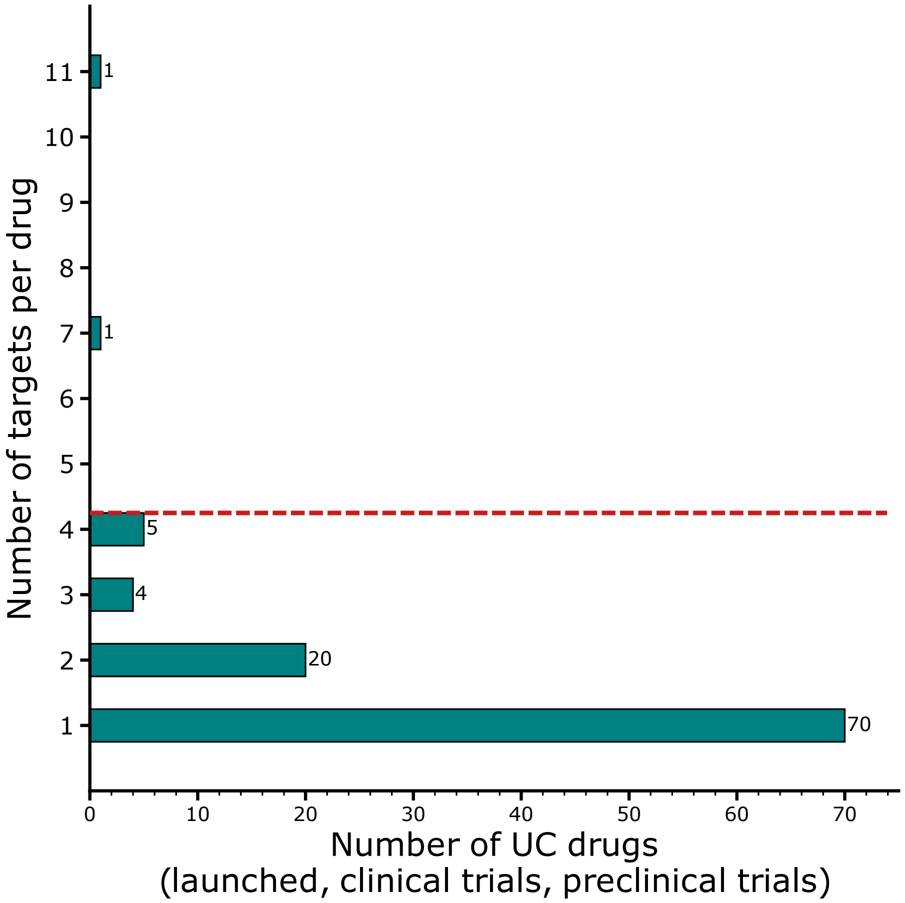

Figure S3: **Number of targets per drug.** The majority of drugs approved or being developed for UC treatment have maximum of 4 simultaneous targets. To avoid promiscuous drugs, we filter out the drugs with > 4 targets in our analysis (red dashed line).

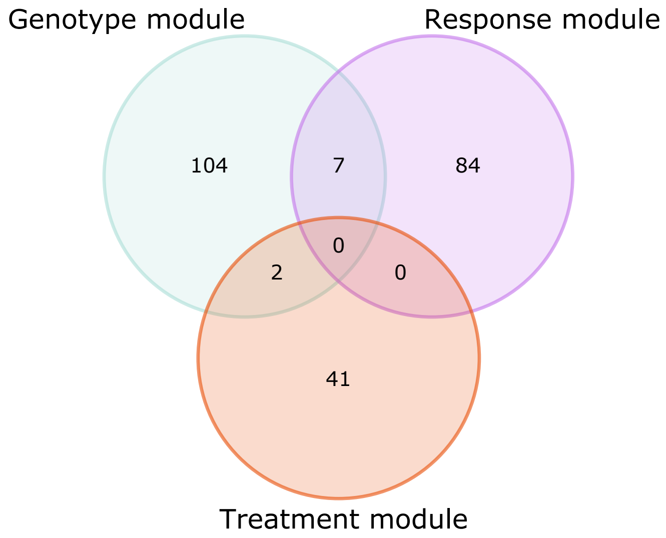

Figure S4: **Venn diagram of the three gene sets corresponding to the UC Genotype, Response, and Treatment modules.**

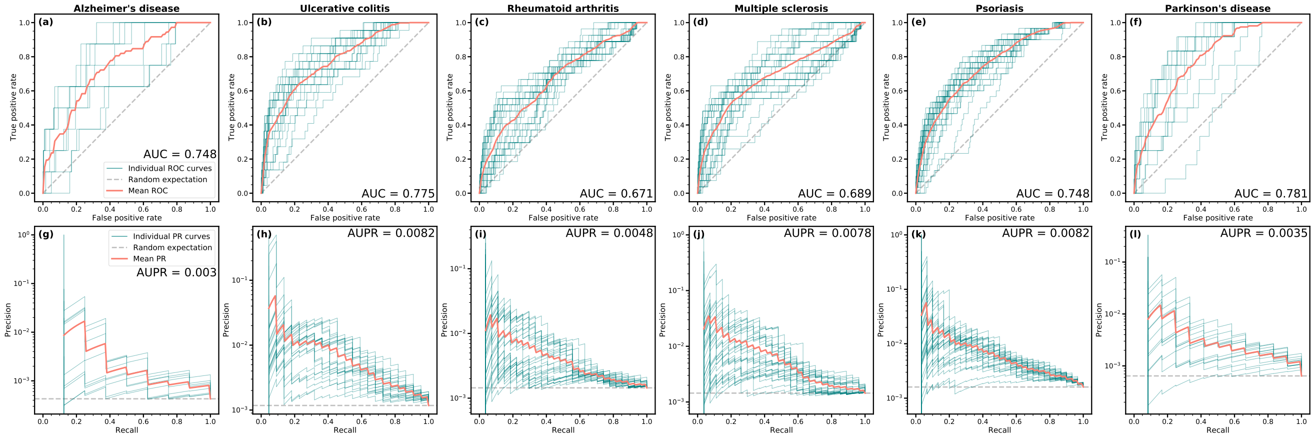

Figure S5: **Recovery of the targets approved for 6 complex disease based on diffusion state distance (DSD).** Receiver operator characteristic (ROC) and Precision-Recall (PR) curves for recovery of know approved targets for treatment of **(a), (g)** Alzheimer's disease; **(b), (h)** ulcerative colitis; **(c), (i)** rheumatoid arthritis; **(d), (j)** multiple sclerosis; **(e), (k)** psoriasis; **(f), (l)** Parkinson's disease. Individual ROC curves demonstrate recovery of the approved targets given one known approved target and DSD from it to the rest of the HI nodes. Red lines represent mean ROC/PR curves obtained by averaging over the individual curves, and area under the curve (AUC/AUPR) is reported for the mean ROC/PR curve.

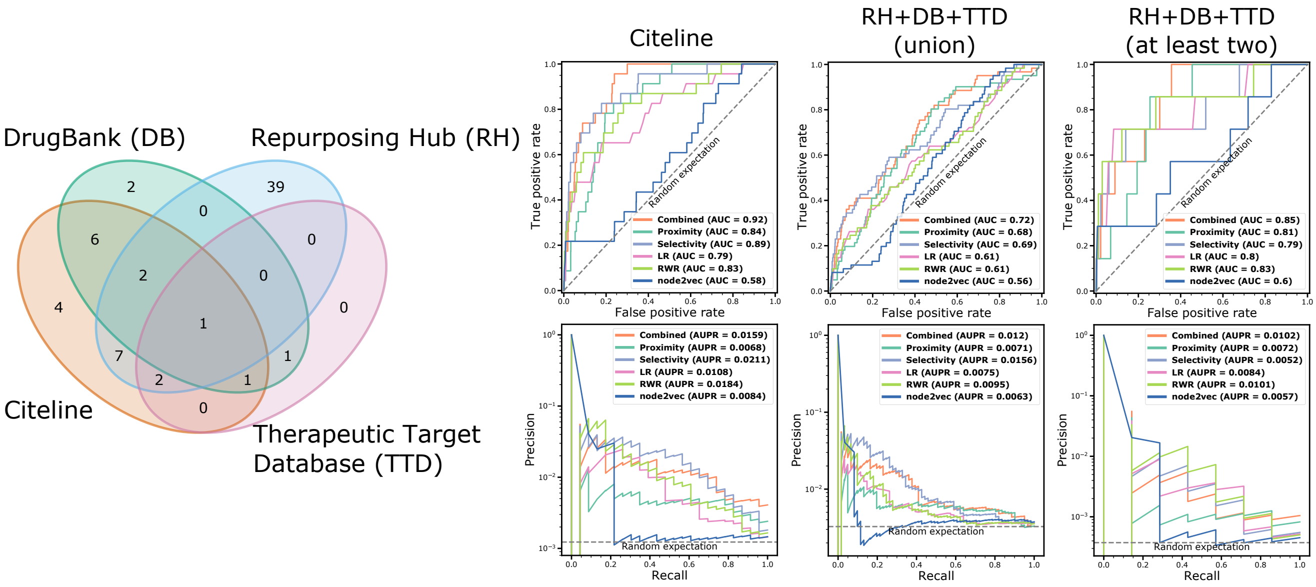

Figure S6: **Venn diagram of the UC approved targets according to different databases and performance curves of recovery of the approved targets.** Recovery of the UC approved targets is tested in three scenarios: (1) considering approved targets from Citeline database; (2) considering the set union of the approved targets from DrugBank, Repurposing Hub, and Therapeutic Target Database; (3) considering the approved targets that are mentioned in at least two of the three open access databases (DrugBank, Repurposing Hub, and Therapeutic Target Database). Areas under the ROC and PR curves are reported in the figures' legends. We note that the seemingly excessive number of targets reported only in Repurposing Hub may be due to the fact that this database only provides a single clinical phase indicator for each drug, and may therefore pull drugs that are reported to be "launched" for some indications, but may still be in clinical or preclinical trials for UC.

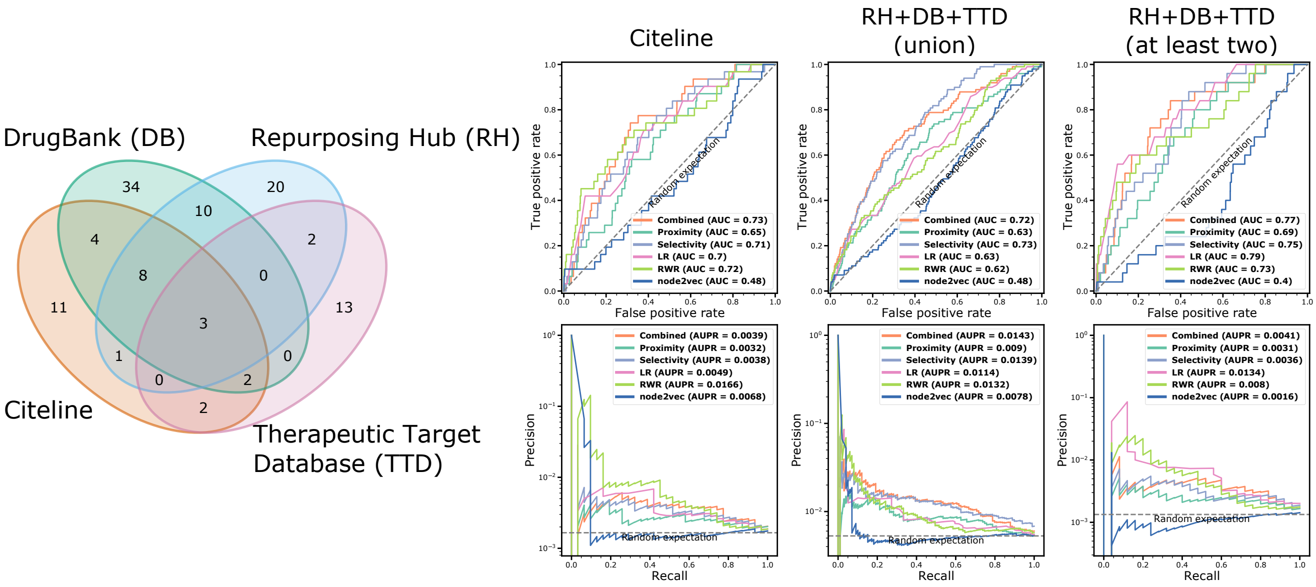

Figure S7: **Venn diagram of the psoriasis approved targets according to different databases and performance curves of recovery of the approved targets.** Recovery of the psoriasis approved targets is tested in three scenarios: (1) considering approved targets from Citeline database; (2) considering the set union of the approved targets from DrugBank, Repurposing Hub, and Therapeutic Target Database; (3) considering the approved targets that are mentioned in at least two of the three open access databases (DrugBank, Repurposing Hub, and Therapeutic Target Database). Areas under the ROC and PR curves are reported in the figures' legends.

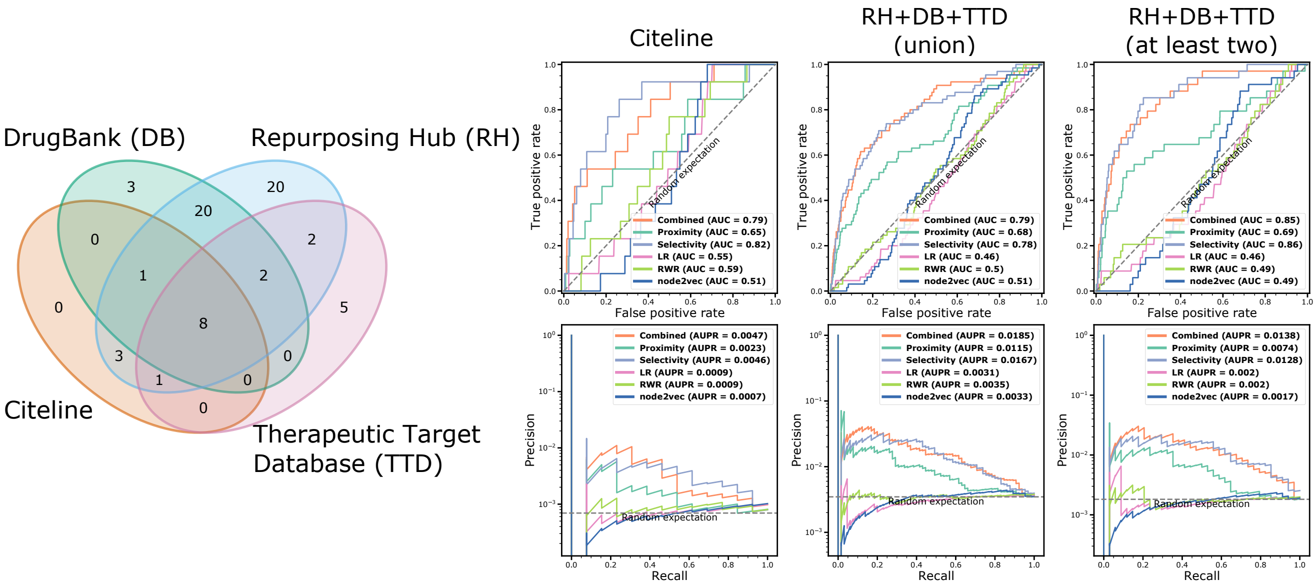

Figure S8: **Venn diagram of the Parkinson's disease (PD) approved targets according to different databases and performance curves of recovery of the approved targets.** Recovery of the PD approved targets is tested in three scenarios: (1) considering approved targets from Citeline database; (2) considering the set union of the approved targets from DrugBank, Repurposing Hub, and Therapeutic Target Database; (3) considering the approved targets that are mentioned in at least two of the three open access databases (DrugBank, Repurposing Hub, and Therapeutic Target Database). Areas under the ROC and PR curves are reported in the figures' legends.

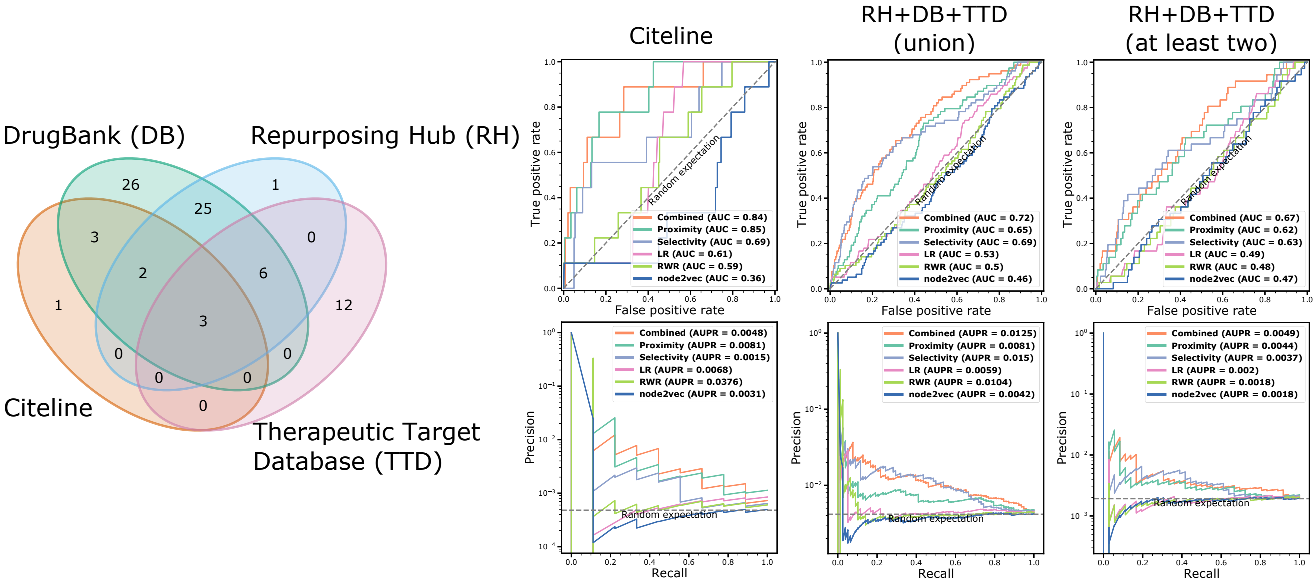

Figure S9: **Venn diagram of the Alzheimer's disease (AD) approved targets according to different databases and performance curves of recovery of the approved targets.** Recovery of the AD approved targets is tested in three scenarios: (1) considering approved targets from Citeline database; (2) considering the set union of the approved targets from DrugBank, Repurposing Hub, and Therapeutic Target Database; (3) considering the approved targets that are mentioned in at least two of the three open access databases (DrugBank, Repurposing Hub, and Therapeutic Target Database). Areas under the ROC and PR curves are reported in the figures' legends.

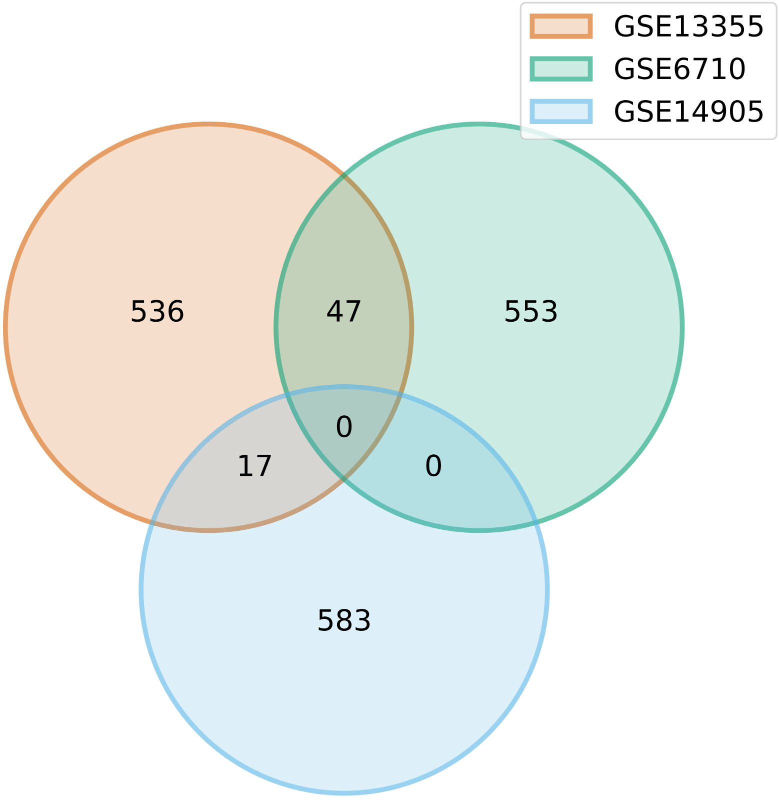

Figure S10: **Venn diagram of the 600 differentially expressed genes in patients with psoriasis with respect to normal controls, according to the three GEO datasets reported in Harmonizome.** Genes that are reported in at least two datasets are retained for the Response module construction.

**GEO accession number**	**Normal controls**	**UC active patients**	**Number of patients/normal controls**	**TNFi response label**	**Response label timepoints**	**Pre-treatment expression data**	**Post-treatment expression data**
**Infliximab, Affymetrix U133 Plus 2 array**							
GSE16879	+	+	24/6	+	week 4-6	+	+
GSE23597	−	+	45/−	+	week 8, 30	+	+
GSE38713	+	+	14/13	−	−	+	−
GSE13367	−	+	8/−	−	−	+	−
GSE36807	+	+	15/7	−	−	+	−
GSE47908	+	+	39/15	−	−	+	−
**Golimumab, Affymetrix U133+ array**							
GSE92415	+	+	87/21	+	week 6	+	+

Table S1: **TNFi treatment studies used to identify a molecular signature of UC patient response.**

GEO accession number	Definition of TNFi response
GSE16879	"For UC and CDc, the response to infliximab was defined as a complete mucosal healing with a decrease of at least 3 points on the histological score for CDc [8] and as a decrease to a Mayo endoscopic subscore of 0 or 1 with a decrease to grade 0 or 1 on the histological score for UC [26,27]. Patients who did not achieve this healing were considered nonresponders although some of them presented endoscopic and/or histologic improvement." [16]
GSE23597	"...defined as a decrease from baseline in the total Mayo score of at least three points and at least 30%, with an accompanying decrease in the subscore for rectal bleeding of at least one point or an absolute subscore for rectal bleeding of 0 or 1 [4]." [17]
GSE92415	"Response was defined as complete mucosal healing and histologic normalization (a Mayo endoscopic subscore of 0 or 1 and a grade of 0 or 1 on the Geboes histological scale)." [18]

Table S2: **Definitions of TNFi response across cohorts with specified UC patients' response labels.**

**Top-K ranked proteins**	**Selectivity ranking**	**Proximity ranking**	**Combined ranking**	**Local radiality ranking**	**Random walk ranking**	**node2vec ranking**
10	0/23	0/23	0/23	0/23	0/23	1/23
50	2/23	1/23	1/23	1/23	3/23	2/23
100	3/23	1/23	3/23	1/23	4/23	3/23
500	11/23	2/23	8/23	8/23	8/23	5/23
$1,000$	14/23	5/23	12/23	10/23	10/23	5/23
$5,000$	19/23	19/23	22/23	15/23	18/23	7/23
$10,000$	22/23	23/23	23/23	20/23	20/23	14/23

Table S3: **Fraction of recovered approved targets for UC treatment according to Citeline database among top-K proteins ranked by selectivity, proximity, combined proximity and selectivity, Local radiality with respect to the Response module, random walk with restart with respect to UC-associated genes, and node2vec embedding ranking with respect to UC-associated genes.**

Drug name	Known mechanism of action
diethylstilbestrol	estrogen receptor agonist
dexamethasone-acetate	glucocorticoid receptor agonist
acarbose	glucosidase inhibitor
betaxolol	adrenergic receptor antagonist
avicin-d	AMP-activated protein kinase activation
piceatannol	SYK inhibitor
calcifediol	vitamin D receptor agonist
UNC-0321	G9a inhibitor
homatropine	acetylcholine receptor antagonist
PD-184352	MEK inhibitor
wortmannin	PI3K inhibitor
ERK-inhibitor-11E	ERK inhibitor
reversine	Aurora kinase inhibitor
vemurafenib	RAF inhibitor
PLX-4720	RAF inhibitor
carbamazepine	carboxamide antiepileptic
leucodin	TNF-alpha, TIMP Metallopeptidase Inhibitor

Table S4: **Drugs and their known mechanisms of action mapped to the protein targets belonging to the Treatment module.**

Pathway identifier	Pathway name	Num. entities found	Num. entities total	Num. interactors found	Num. interactors total	Entities ratio	Entities p-val.	Entities FDR	Num. reactions found	Num. reactions total	Reactions ratio
R-HSA-6783783	Interleukin-10 signaling	16	86	6	93	5.7E-03	4.5E-09	5.4E-06	15	15	1.1E-03
R-HSA-383280	Nuclear Receptor transcription pathway	13	86	1	34	5.7E-03	9.1E-09	5.6E-06	2	2	1.4E-04
R-HSA-6785807	Interleukin-4 and Interleukin-13 signaling	19	211	9	162	1.4E-02	8.3E-07	3.4E-04	44	47	3.3E-03
R-HSA-912526	Interleukin receptor SHC signaling	6	29	0	3	1.9E-03	6.5E-06	1.6E-03	6	6	4.3E-04
R-HSA-5660668	CLEC7A/inflammasome pathway	5	8	2	25	5.3E-04	6.7E-05	1.4E-02	3	4	2.8E-04
R-HSA-449147	Signaling by Interleukins	58	658	53	2161	4.3E-02	1.4E-04	2.3E-02	352	505	3.6E-02
R-HSA-8877330	RUNX1 and FOXP3 control the development of regulatory T lymphocytes (Tregs)	6	17	3	42	1.1E-03	1.6E-04	2.4E-02	15	20	1.4E-03
R-HSA-2219530	Constitutive Signaling by Aberrant PI3K in Cancer	7	96	0	0	6.3E-03	4.1E-04	5.2E-02	2	2	1.4E-04
R-HSA-448706	Interleukin-1 processing	4	12	2	16	7.9E-04	4.3E-04	5.2E-02	4	5	3.6E-04
R-HSA-9027276	Erythropoietin activates Phosphoinositide-3-kinase (PI3K)	4	16	1	12	1.1E-03	5.7E-04	6.3E-02	5	7	5.0E-04
R-HSA-9020958	Interleukin-21 signaling	3	12	0	1	7.9E-04	8.2E-04	8.2E-02	5	5	3.6E-04
R-HSA-9012546	Interleukin-18 signaling	3	11	0	5	7.2E-04	1.0E-03	9.4E-02	4	4	2.8E-04
R-HSA-5632681	Ligand-receptor interactions	3	8	0	9	5.3E-04	1.2E-03	1.1E-01	4	4	2.8E-04
R-HSA-9671555	Signaling by PDGFR in disease	4	27	0	10	1.8E-03	1.8E-03	1.5E-01	8	24	1.7E-03
R-HSA-9673770	Signaling by PDGFRA extracellular domain mutants	3	19	0	0	1.3E-03	2.4E-03	1.7E-01	3	7	5.0E-04
R-HSA-9673767	Signaling by PDGFRA transmembrane, juxtamembrane and kinase domain mutants	3	19	0	0	1.3E-03	2.4E-03	1.7E-01	3	7	5.0E-04
R-HSA-3928663	EPHA-mediated growth cone collapse	4	33	0	11	2.2E-03	3.4E-03	2.2E-01	3	4	2.8E-04
R-HSA-1963642	PI3K events in ERBB2 signaling	3	22	0	0	1.4E-03	3.6E-03	2.2E-01	7	7	5.0E-04
R-HSA-8853659	RET signaling	7	43	5	110	2.8E-03	3.6E-03	2.2E-01	23	24	1.7E-03
R-HSA-418990	Adherens junctions interactions	7	35	1	116	2.3E-03	3.9E-03	2.3E-01	2	16	1.1E-03
R-HSA-1280215	Cytokine Signaling in Immune system	69	1107	67	2946	7.3E-02	4.2E-03	2.3E-01	456	726	5.2E-02
R-HSA-109704	PI3K Cascade	5	58	1	24	3.8E-03	5.5E-03	2.7E-01	3	6	4.3E-04
R-HSA-5603029	IkBA variant leads to EDA-ID	2	8	0	0	5.3E-04	5.6E-03	2.7E-01	2	2	1.4E-04
R-HSA-194313	VEGF ligand-receptor interactions	3	8	4	26	5.3E-04	7.7E-03	3.2E-01	4	4	2.8E-04
R-HSA-195399	VEGF binds to VEGFR leading to receptor dimerization	3	8	4	26	5.3E-04	7.7E-03	3.2E-01	3	3	2.1E-04
R-HSA-112399	IRS-mediated signalling	5	65	1	24	4.3E-03	7.7E-03	3.2E-01	4	9	6.4E-04
R-HSA-6811558	PI5P, PP2A and IER3 Regulate PI3K/AKT Signaling	8	129	1	85	8.5E-03	8.6E-03	3.4E-01	4	7	5.0E-04
R-HSA-8854691	Interleukin-20 family signaling	8	29	15	193	1.9E-03	9.6E-03	3.4E-01	52	56	4.0E-03
R-HSA-3928665	EPH-ephrin mediated repulsion of cells	5	55	0	39	3.6E-03	9.6E-03	3.4E-01	8	9	6.4E-04
R-HSA-9028335	Activated NTRK2 signals through PI3K	2	11	0	0	7.2E-04	1.0E-02	3.4E-01	2	2	1.4E-04
R-HSA-2428928	IRS-related events triggered by IGF1R	5	69	2	30	4.5E-03	1.1E-02	3.4E-01	5	12	8.5E-04
R-HSA-9670439	Signaling by phosphorylated juxtamembrane, extracellular and kinase domain KIT mutants	3	28	1	8	1.8E-03	1.3E-02	3.4E-01	7	11	7.8E-04
R-HSA-9669938	Signaling by KIT in disease	3	28	1	8	1.8E-03	1.3E-02	3.4E-01	7	26	1.8E-03
R-HSA-2428924	IGF1R signaling cascade	5	72	2	30	4.7E-03	1.3E-02	3.4E-01	5	17	1.2E-03
R-HSA-2404192	Signaling by Type 1 Insulin-like Growth Factor 1 Receptor (IGF1R)	5	73	2	30	4.8E-03	1.3E-02	3.4E-01	5	19	1.3E-03
R-HSA-9700649	Drug resistance of ALK mutants	1	1	0	0	6.6E-05	1.4E-02	3.4E-01	7	7	5.0E-04
R-HSA-9717319	brigatinib-resistant ALK mutants	1	1	0	0	6.6E-05	1.4E-02	3.4E-01	1	1	7.1E-05
R-HSA-9717301	NVP-TAE684-resistant ALK mutants	1	1	0	0	6.6E-05	1.4E-02	3.4E-01	1	1	7.1E-05
R-HSA-9717323	ceritinib-resistant ALK mutants	1	1	0	0	6.6E-05	1.4E-02	3.4E-01	1	1	7.1E-05
R-HSA-5602410	TLR3 deficiency - HSE	1	1	0	0	6.6E-05	1.4E-02	3.4E-01	1	1	7.1E-05
R-HSA-9717316	alectinib-resistant ALK mutants	1	1	0	0	6.6E-05	1.4E-02	3.4E-01	1	1	7.1E-05
R-HSA-9717326	crizotinib-resistant ALK mutants	1	1	0	0	6.6E-05	1.4E-02	3.4E-01	1	1	7.1E-05
R-HSA-9717329	lorlatinib-resistant ALK mutants	1	1	0	0	6.6E-05	1.4E-02	3.4E-01	1	1	7.1E-05
R-HSA-9717264	ASP-3026-resistant ALK mutants	1	1	0	0	6.6E-05	1.4E-02	3.4E-01	1	1	7.1E-05
R-HSA-5673001	RAF/MAP kinase cascade	13	322	2	183	2.1E-02	1.6E-02	3.9E-01	26	75	5.3E-03
R-HSA-190371	FGFR3b ligand binding and activation	2	10	0	4	6.6E-04	1.6E-02	3.9E-01	2	2	1.4E-04
R-HSA-2129379	Molecules associated with elastic fibres	3	37	0	2	2.4E-03	1.7E-02	4.0E-01	3	10	7.1E-04

R-HSA-199418	Negative regulation of the PI3K/AKT network	8	137	1	109	9.0E-03	1.8E-02	4.0E-01	4	10	7.1E-04
R-HSA-1250342	PI3K events in ERBB4 signaling	2	15	0	0	9.9E-04	1.8E-02	4.2E-01	2	2	1.4E-04
R-HSA-1810476	RIP-mediated NFkB activation via ZBP1	3	19	1	26	1.3E-03	2.1E-02	4.7E-01	4	4	2.8E-04
R-HSA-9006335	Signaling by Erythropoietin	4	35	2	48	2.3E-03	2.3E-02	5.1E-01	6	25	1.8E-03
R-HSA-1839124	FGFR1 mutant receptor activation	5	39	10	82	2.6E-03	2.3E-02	5.2E-01	9	25	1.8E-03
R-HSA-5602566	TICAM1 deficiency - HSE	1	2	0	0	1.3E-04	2.7E-02	5.7E-01	1	1	7.1E-05
R-HSA-1839122	Signaling by activated point mutants of FGFR1	2	15	0	4	9.9E-04	2.8E-02	6.0E-01	2	4	2.8E-04
R-HSA-5655332	Signaling by FGFR3 in disease	4	33	3	56	2.2E-03	3.2E-02	6.5E-01	13	21	1.5E-03
R-HSA-5684996	MAPK1/MAPK3 signaling	14	329	3	296	2.2E-02	3.3E-02	6.5E-01	31	82	5.8E-03
R-HSA-190375	FGFR2c ligand binding and activation	2	17	0	4	1.1E-03	3.4E-02	6.5E-01	2	2	1.4E-04
R-HSA-1839130	Signaling by activated point mutants of FGFR3	2	17	0	4	1.1E-03	3.4E-02	6.5E-01	2	6	4.3E-04
R-HSA-2033514	FGFR3 mutant receptor activation	2	17	0	4	1.1E-03	3.4E-02	6.5E-01	2	10	7.1E-04
R-HSA-421270	Cell-cell junction organization	7	68	1	165	4.5E-03	3.5E-02	6.6E-01	2	21	1.5E-03
R-HSA-9014826	Interleukin-36 pathway	2	7	0	15	4.6E-04	3.7E-02	7.1E-01	3	3	2.1E-04
R-HSA-190373	FGFR1c ligand binding and activation	2	15	0	8	9.9E-04	4.0E-02	7.2E-01	2	3	2.1E-04
R-HSA-210993	Tie2 Signaling	5	23	7	123	1.5E-03	4.2E-02	7.4E-01	14	14	9.9E-04
R-HSA-2559582	Senescence-Associated Secretory Phenotype (SASP)	5	91	2	49	6.0E-03	4.3E-02	7.4E-01	8	22	1.6E-03
R-HSA-8851907	MET activates PI3K/AKT signaling	2	10	1	15	6.6E-04	4.3E-02	7.4E-01	4	5	3.6E-04
R-HSA-190322	FGFR4 ligand binding and activation	2	17	0	7	1.1E-03	4.3E-02	7.4E-01	2	4	2.8E-04
R-HSA-190372	FGFR3c ligand binding and activation	2	18	0	6	1.2E-03	4.3E-02	7.4E-01	2	4	2.8E-04
R-HSA-190239	FGFR3 ligand binding and activation	2	19	0	6	1.3E-03	4.7E-02	7.7E-01	4	6	4.3E-04
R-HSA-5654710	PI-3K cascade:FGFR3	4	24	10	78	1.6E-03	4.8E-02	7.7E-01	7	7	5.0E-04
R-HSA-5654720	PI-3K cascade:FGFR4	4	25	10	78	1.6E-03	5.0E-02	8.0E-01	7	7	5.0E-04

Table S5: **Pathways enriched by the UC prioritized targets derived from Reactome.**

Supplementary Information References

[SI1] Rouillard, A. D. et al. The harmonizome: a collection of processed datasets gathered to serve and mine knowledge about genes and proteins. Database **2016** (2016).

[SI2] Xicoy, H., Wieringa, B. & Martens, G. J. The sh-sy5y cell line in parkinson's disease research: a systematic review. Molecular neurodegeneration **12**, 1–11 (2017).

[SI3] Kanehisa, M. & Goto, S. Kegg: kyoto encyclopedia of genes and genomes. Nucleic acids research **28**, 27–30 (2000).

[SI4] Kuleshov, M. V. et al. Enrichr: a comprehensive gene set enrichment analysis web server 2016 update. Nucleic acids research **44**, W90–W97 (2016).

[SI5] Cojocaru, M., Cojocaru, I. M. & Silosi, I. Multiple autoimmune syndrome. Maedica **5**, 132 (2010).

[SI6] Anaya, J.-M., Rojas-Villarraga, A. & García-Carrasco, M. The autoimmune tautology: from polyautoimmunity and familial autoimmunity to the autoimmune genes (2012).

[SI7] Shapiro, J. M. et al. Immunoglobulin a targets a unique subset of the microbiota in inflammatory bowel disease. Cell Host & Microbe **29**, 83–93 (2021).

[SI8] van Kessel, K. P., Bestebroer, J. & van Strijp, J. A. Neutrophil-mediated phagocytosis of staphylococcus aureus. Frontiers in immunology **5**, 467 (2014).

[SI9] Bassetti, S. et al. Staphylococcus aureus in patients with rheumatoid arthritis under conventional and anti-tumor necrosis factor-alpha treatment. The Journal of rheumatology **32**, 2125–2129 (2005).

[SI10] O'neill, L. A., Golenbock, D. & Bowie, A. G. The history of toll-like receptors—redefining innate immunity. Nature Reviews Immunology **13**, 453–460 (2013).

[SI11] Hua, Z. & Hou, B. Tlr signaling in b-cell development and activation. Cellular & molecular immunology **10**, 103–106 (2013).

[SI12] Sánchez-Muñoz, F. et al. Transcript levels of toll-like receptors 5, 8 and 9 correlate with inflammatory activity in ulcerative colitis. BMC gastroenterology **11**, 1–9 (2011).

[SI13] Kinugasa, T., Sakaguchi, T., Gu, X. & Reinecker, H.-C. Claudins regulate the intestinal barrier in response to immune mediators. Gastroenterology **118**, 1001–1011 (2000).

[SI14] Maloy, K. & Kullberg, M. Il-23 and th17 cytokines in intestinal homeostasis. Mucosal immunology **1**, 339–349 (2008).

[SI15] Bischoff, S. C. et al. Intestinal permeability–a new target for disease prevention and therapy. BMC gastroenterology **14**, 1–25 (2014).

[SI16] Arijs, I. et al. Mucosal gene expression of antimicrobial peptides in inflammatory bowel disease before and after first infliximab treatment. PloS one **4**, e7984 (2009).

[SI17] Toedter, G. et al. Gene expression profiling and response signatures associated with differential responses to infliximab treatment in ulcerative colitis. Official journal of the American College of Gastroenterology— ACG **106**, 1272–1280 (2011).

[SI18] Telesco, S. E. et al. Gene expression signature for prediction of golimumab response in a phase 2a open-label trial of patients with ulcerative colitis. Gastroenterology **155**, 1008–1011 (2018).
